# Improved Prediction of Aqueous Solubility of Novel Compounds by Going Deeper With Deep Learning

**DOI:** 10.3389/fonc.2020.00121

**Published:** 2020-02-11

**Authors:** Qiuji Cui, Shuai Lu, Bingwei Ni, Xian Zeng, Ying Tan, Ya Dong Chen, Hongping Zhao

**Affiliations:** ^1^School of Science, China Pharmaceutical University, Nanjing, China; ^2^Department of Biological Medicine, School of Pharmacy, Fudan University, Shanghai, China; ^3^The State Key Laboratory of Chemical Oncogenomics, Key Laboratory of Chemical Biology, Shenzhen Technology and Engineering Laboratory for Personalized Cancer Diagnostics and Therapeutics, The Graduate School at Shenzhen, Shenzhen Kivita Innovative Drug Discovery Institute, Tsinghua University, Shenzhen, China

**Keywords:** aqueous solubility, deep learning, artificial intelligence, compounds, chemical, anti-cancer drug discovery

## Abstract

Aqueous solubility is an important physicochemical property of compounds in anti-cancer drug discovery. Artificial intelligence solubility prediction tools have scored impressive performances by employing regression, machine learning, and deep learning methods. The reported performances vary significantly partly because of the different datasets used. Solubility prediction on novel compounds needs to be improved, which may be achieved by going deeper with deep learning. We constructed deeper-net models of ~20-layer modified ResNet convolutional neural network architecture, which were trained and tested with 9,943 compounds encoded by molecular fingerprints. Retrospectively tested by 62 recently-published novel compounds, one deeper-net model outperformed four established tools, shallow-net models, and four human experts. Deeper-net models also outperformed others in predicting the solubility values of a series of novel compounds newly-synthesized for anti-cancer drug discovery. Solubility prediction may be improved by going deeper with deep learning. Our deeper-net models are accessible at http://www.npbdb.net/solubility/index.jsp.

## Introduction

Aqueous solubility is an important physicochemical property of compounds in anti-cancer drug discovery and development, impacting pharmacokinetic properties and formulations (1, 2). To facilitate solubility assessment, a number of artificial intelligence (AI) solubility prediction tools have been developed by employing regression and modeling (3, 4), machine learning (5–9), and deep learning (10–12) methods. These tools have scored impressive performances with high R^2^ (e.g., 0.62–0.97) and low RMSE (e.g., 0.29–0.89) values (5, 13). However, the reported performances vary significantly, even among the same tools, partly because of the different datasets used. For instance, the reported R^2^ and RMSE values of MOE software V2010.10 are 0.62 and 0.51 (8) and those in a 2014 publication are 0.27 and 1.05 (14). The reported R^2^ and RMSE values of QikProp software V1.6, V2.1, and V3.2 are 0.9 and 0.8 (6), 0.95 and 0.63 (15), and 0.45 and 0.86 (8), respectively.

AI solubility prediction tools may be critically tested by newly-published novel compounds. Tested by 62 novel compounds published since November 2017 (Methods section), four established tools MOE V2016.0802, QikProp QP18 and CIQP18, and AlogGPS V2.1 scored significantly lower R^2^ (<0.2) and higher RMSE (0.814–1.162) values (Results section) than the typically-reported values (5, 6, 8, 14, 15). Our own-developed deep learning model of typically-employed shallow-net architecture (Methods section), trained and tested with 9,943 compounds, also scored lower R^2^ (0.307) and higher RMSE (0.739) values (Results section). Hence, there is a need for improved solubility prediction particularly on novel compounds to promote oral anti-cancer drug development. In AI field, deep learning methods with distinguished learning capabilities (16) [which has been proved by prediction of CRISPR-Cpf1 guide RNA activity (17) and prediction of protein-ligand binding affinity (18)] are useful for this task, but their potential has yet to be fully realized.

The published deep learning solubility prediction models are primarily shallow-nets (3–7 layers) (10–12). Deep learning performances have been routinely enhanced by going deeper (adding more layers to shallow-nets) (19–21). Although performances can also be enhanced by going wider (22), it may be practically easier to develop deeper-nets by tapping into the well-established architectures that require fewer parameters (19–21). The depth of deeper-nets or the width of wider-nets is constrained by the limited number of compounds with experimental solubility data. The architecture with fewer parameters, convolutional neural networks (CNN), is therefore preferred. A question is whether the superior local-feature learning capability of CNN can adequately learn molecular features of compounds. To fit with the local-feature learning capability of CNN, compounds are better represented by substructure-encoded molecular fingerprints (23) instead of molecular descriptors used for solubility prediction by previously-developed deep learning models (10–12). Molecular fingerprints are vectors with individual components encoding specific sub-structures of molecules. Hence, the superior local-feature learning capability of CNN is expected to be useful for capturing the key sub-structural elements and their combinations contributing the solubility of molecules.

We constructed N-layer CNN models (*N* = 14, 20, and 26) using 9,943 compounds and based on a residual network (ResNet) architecture (20), which are significantly deeper than the previously-developed 3–7 layers shallow-net models (10–12). The solubility prediction capability of our deeper-net models was tested by retrospective prediction of the experimental solubility of 62 recently-published novel compounds beyond the training and testing compounds. These performances were compared with those of four established tools, shallow-net models and four human experts. Our deeper-net models and others were further tested by a real anti-cancer drug discovery project with a series of novel compounds newly-synthesized for discovering FLT3 inhibitors. These compounds were considered difficult for solubility estimation by medicinal chemistry experts, which are ideal for rigorous test of solubility prediction models. Our models are accessible at http://www.npbdb.net/solubility/index.jsp for supporting broader tests.

## Materials and Methods

### Data Collection and Processing

**Figure 1:**
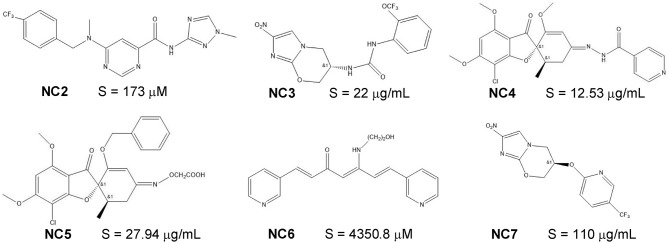
The molecular structures and experimental solubility S values of six recently-published novel compounds.

A total of 10,166 compounds with experimental aqueous solubility value were collected from ChemIDplus database (24) and Pubmed (9, 25, 26) literature search up to November 2017. Another 62 recently-published novel compounds with experimental aqueous solubility value (Supplementary Figure 1, 6 representative compounds in Figure 1) were collected from PMC database (27–31) search using keyword combination of “novel”, “new,” and “solubility” and under the following criteria: published between November 2017 and May 2018, and solubility measured at room-temperature and around pH 7.0. For the 10,166 compounds, their SMILES strings (which encode sub-structures), InChIKeys (chemical structure identifiers) and aqueous solubility values were collected from the searched sources. For the 62 novel compounds, their structures were drawn from literature-reported structures by using ChemDraw 18.0 and then converted to the SMILES strings by using RDKit^1^. Solubility S values in different units (e.g., μg/mL, mg/mL, and mg/L) were converted to mol/L and transformed into logS (in logarithmic units) values. The SMILES strings were converted to canonical SMILES strings for consistency by using Open Babel (32). Duplicates were removed by InChIKeys comparisons. The canonical SMILES of the remaining non-redundant 9,943 compounds (Supplementary Table 1, the basic physical properties detailed in Supplementary Table 2) and the 62 novel compounds were converted into the Pubchem molecular fingerprints (which encode sub-structures by 881 bits) using PaDEL (33).

### Established Tools and a Deep Learning Model of Typically-Employed Shallow-Net Architecture for Solubility Prediction

Solubility prediction performances were comparatively evaluated with respect to four established software tools [MOE V2016.0802^2^, QikProp 2018-4 QP18 and CIQP18^3^, and AlogGPS V2.1 based on an artificial neural network method (5)]. The deep learning model was developed based on a typically-employed shallow-net deep neural network (DNN) architecture for solubility prediction (11), which is a 4 hidden-layers DNN (Supplementary Figure 2) with the network architecture and parameter sets re-constructed based on the literature descriptions (11) with the following minor variations: the activation function was changed from SReLU to ReLU and the compounds were represented by pubchem molecular fingerprints instead of fp6 molecular fingerprints. The numbers of nodes of the hidden layers are 512, 1,024, 2,048, and 4,096. The parameters of L2 regularization and dropout regularization are 0.001 and 0.5. The 9,943 compounds were randomly divided into 90% training and 10% testing datasets for training the DNN model.

### Development of Deep learning Models of Deeper-Net Architecture for Solubility Prediction

**Figure 2:**
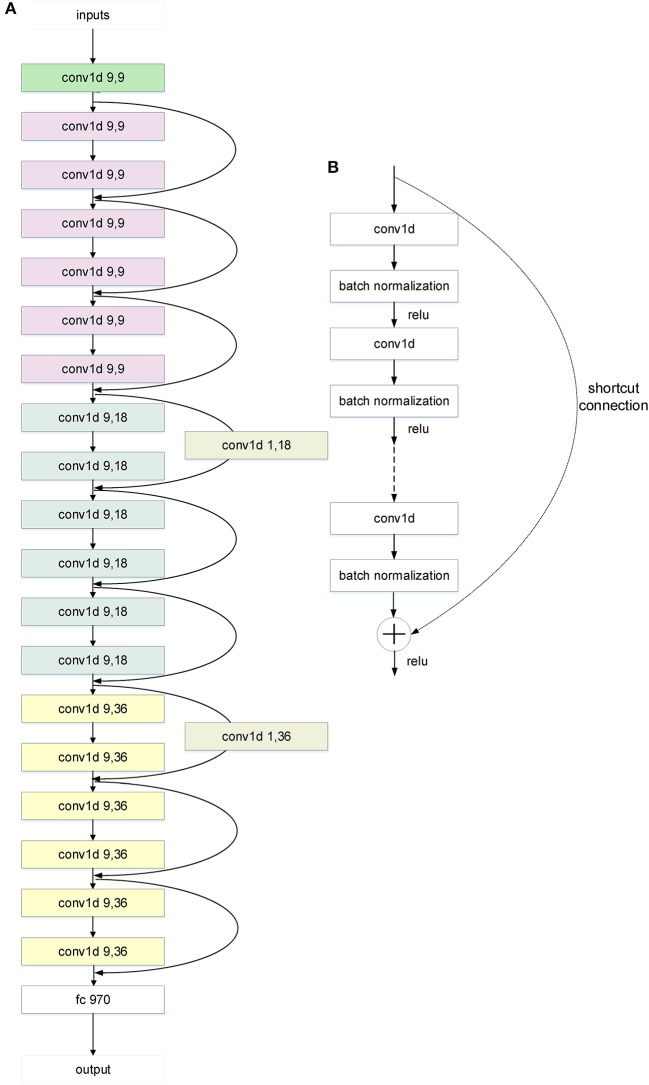
The architecture of the 20-layer CNN ResNet-like deep learning model. **(A)** A CNN ResNet-like deep learning model with 20 parameter layers. The “conv1d x,y” is a 1D convolution layer with x kernel sizes and y filters. And the curvy arrows are the shortcut connections. The shortcut connection with a parameter layer increases dimensions. The different color means different layer class in the architecture. “Green” means the first layer, “white” means the last layer, “gray” means the parameter layer of the shortcut connection, and the others mean the residual layers. The color change of the residual layers from purple to blue to yellow indicates the tensor dimension change from 9 to 18 to 36. **(B)** The shortcut connection in the architecture of CNN ResNet-like deep learning model. Shortcut connections simply perform identity mapping by skipping one or more layers (20). Their outputs are added to the outputs of the stacked layers without extra parameter and computational complexity.

The deeper-net models were based on the ResNet architecture (20) with the usual matrix forms of the ResNet layers, filters and feature maps replaced by vector forms. The numbers of layers N are 14, 20 (Figure 2), and 26 (Supplementary Figure 3) (N-1 CNN layers and 1 fully-connected layer). The vector forms were used because the inputs are 881-dimensional vectors (Pubchem fingerprints) instead of matrices of image pixel values. These CNN models were trained by the 10-fold cross validation method used for the development of two shallow-net deep learning solubility prediction models (10, 12). In the 10-fold cross validation method, the 9,943 compounds were randomly divided into 10 sets of approximately equal sizes, with each set used once as a testing dataset, and the remaining 9 sets as training dataset for training the CNN models. The CNN hyperparameters were optimized based on the overall performance of the 10 training/testing datasets. These hyperparameters include loss function, kernel sizes, number of filters, stride lengths, number of fully-connected hidden layers, number of neurons of the fully-connected layer, activation function, optimizer, learning rate, weight initialization, regularization, batch size, and epochs. Multiple activation functions (Sigmoid, ReLU, Softmax) were evaluated in both activation layers and the activation arguments of all forward layers. The weight initialization was uniform. L2 regularization was added by small amounts of L2 weight decay. A solubility value regression model was trained by least squares fit $\left(-R^{2}=-(1-(\sum_{i=0}^{n-1}{\left(y_{i}-{\hat{y}}_{i}\right)}^{2}/\sum_{i=0}^{n-1}{\left(y_{i}-\bar{y}\right)}^{2}))\right)$ and experimental (*y*_*i*_) solubility values of the n training compounds as the loss function of the output of our deeper-net models.

### Performance Evaluation Metrics

The solubility prediction performances of the developed deep learning models were assessed by two metrics used in the evaluation of previously-developed shallow-net deep learning models (10, 12). One is the R^2^ value, where R is the Pearson correlation coefficient defined by:

$$\begin{array}{l} R^{2}=1-\frac{\sum_{i=0}^{n-1}{\left(y_{i}-\hat{y_{i}}\right)}^{2}}{\sum_{i=0}^{n-1}{\left(y_{i}-\bar{y}\right)}^{2}} \end{array}$$

The second is the root mean squared error RMSE defined by:

$$\begin{array}{l} \text{RMSE}=\sqrt{\frac{\sum_{i=0}^{n-1}{\left(y_{i}-\hat{y_{i}}\right)}^{2}}{n}} \end{array}$$

where $\hat{y_{i}}\;$is the predicted and *y*_*i*_ is experimental solubility values of the training compounds.

In statistics, R^2^, the coefficient of determination, is the proportion of the variance in the dependent variable that is predictable from the independent variable(s). It is a statistical measure used in a regression model to indicate that how well the model fits the data. Theoretically, it denotes a goodness-of-fit indicator that can vary from –∞ to 1. The closer the R^2^ value is to 1, the better the model fits the data, and vice versa. The other metric, RMSE, is the square root of the average of squared errors. It is a statistical measure of the differences between the values predicted by a model and the true values. RMSE is always non-negative, and the value closer to 0 indicates the better fit to the data.

### Chemical Synthesis and Experimental Aqueous Solubility Determination

In one of our drugs discovering projects toward antitumor therapeutics, a series of novel FLT3 inhibitors were designed and synthesized using the structure-based drug design methods. The aqueous solubilities (pH = 7) of these compounds were measured using the modified shake flask method and RP-HPLC (34, 35). Each compound was added into a 1.5 mL Eppendorf tube containing Milli-Q water (1 mL) to form the precipitates at 25°C. Then the mixture was subjected to a solubility-equilibrium stage. The tube was shook at 300 rpm at 25°C for 24 h. The precipitate was separated by centrifugation at 23,000 g for 20 min. Subsequently, 0.25 mL of supernatant was transferred into a 1 mL Eppendorf tube, and it was centrifuged again with the same settings used above. The supernatant was then used for HPLC analysis. An Agilent 1260 Infinity LC system (Agilent Technologies, Inc., Santa Clara, California) was used. For HPLC conditions, a ZORBAX SB-C18 column (5 μM, 4.6 × 150 mm; Agilent), a flow rate of 0.8 mL/min for mobile phase, a UV wavelength of 250 nM and a column temperature of 30°C were used. The sample was injected automatically by a mechanical arm and separated by a constant mixture of methanol/PBS (pH 5.6), 90:10. For each compound, a standard curve consisting of four concentrations was established. The synthetic methods of all but compound SC5 and SC6 have been published in literatures (36–39). The synthetic methods of SC5 and SC6 are described in Supplementary Method 1.

## Results

### The Training of the Deeper-Net Models and Solubility Prediction Performance Evaluation

Using 9,943 compounds and 10-fold cross validation method, three deeper-net models of 14-, 20-, and 26-layer were developed. The ranges and the optimal hyperparameter values for the 20-layer model (which is the top performing model based on the loss function R^2^ values) are given in Supplementary Table 3. The 10-fold cross validation performances of the 14-, 20-, and 26-layer models are R^2^ = 0.72–0.78, 0.74–0.79, and 0.72–0.79, and RMSE = 0.988–1.144, 1.006–1.112, 1.015–1.151, respectively (detailed in Supplementary Table 4). In spite of different depths, these models performed similarly well, possibly because the superior predictive capability of these deeper-net models cannot be fully tested by 1-fold (1/10) testing datasets. The test by novel compounds may be better for probing the predictive capabilities. The reported 10-fold cross validation performances of the two previously-developed shallow-net models are R^2^ = 0.86–0.92 and 0.90–0.92, and RMSE = 0.58–0.79 and 0.45–0.50, respectively (10, 12), which are substantially better than those of our deeper-net models. It is noted that our datasets (testing 994 compounds, training 8,949 compounds) are significantly larger than those of the two previously-developed shallow-net models (testing 102–287 and 129–154 compounds, training 923–2,586 and 1,161–1,537 compounds, respectively) (10, 12). Caution is needed in a direct comparison of the performance statistics of these models. The significantly more diverse testing datasets may partly contribute to the lower performance statistics. But the more diverse training datasets likely lead to more robust prediction capability than the less diverse training datasets. Because of the inaccessibility of the previously-published shallow-net models, it is impossible to test these models on a common set of diverse compounds. Therefore, these models were tested on the 62 newly-published novel compounds and a series of novel compounds from our anti-cancer drug discovery project with solubility measured for the first time in this work.

### Prediction of the Solubility Values of Literature-Reported Novel Compounds by the Deeper-Net Models in Comparison With the Established Tools and Shallow-Net Models

The solubility prediction capability of our deeper-net models was tested by the 62 newly-published novel compounds. We also trained 1-layer DNN model, 6-layer DNN model, and 8-layer ResNet-like model as our shallow-net models. The testing results of these models are included in Table 1, and the predicted logS values of these models with respect to experimental logS values are in Supplementary Table 5. Based on the R^2^ and RMSE values, the 20-layer deeper-net model (R^2^ = 0.412, RMSE = 0.681) performed substantially better than all the other models including the four established tools and the shallow-net models (R^2^ in the range of <0.2 to 0.307, RMSE = 0.739–0.982). The R^2^ and RMSE values of four established tools, shallow-net and deeper-net deep learning models were evaluated by the bootstrap sampling method. The mean, standard deviation and 95% confidence interval of R^2^ and RMSE values for 10,000 bootstrap samples of 62 recently-published novel compounds were detailed in Supplementary Table 6. Judged by the percent of predicted logS values within 10-fold of experimental value, all but one model achieved high performances (66.1%), suggesting the usefulness of both established tools and deep learning models for accessing solubility categories. Nonetheless, the 20-layer deeper-net model substantially outperforms all other models. These suggested that going deeper with deep learning at appropriate depth may give rise to significantly improved solubility prediction on novel compounds. The lower R^2^ and RMSE values of the 26-layer model (R^2^ = 0.075, RMSE = 0.854) over the 20-layer model indicated signs of overfitting in going further deeper beyond ~20-layer.

**Table 1:** Performance on the logS prediction of 62 recently-published novel compounds^a^.

**Model**	**R^**2**^**	**RMSE**	**PCT-10-fold^b^ (%)**
**Established tools**			
MOE V2016.0802	<0.2	0.908	74.2
QikProp 2018-4 QP18	<0.2	0.926	69.4
QikProp 2018-4 CIQP18	<0.2	1.162	54.8
AlogGPS V2.1	0.160	0.814	77.4
**Shallow-net deep learning model of a**			
**typically-employed architecture for**			
**solubility prediction**			
4-layer DNN model	0.307	0.739	80.7
**Shallow-net deep learning models developed**			
**in this work**			
1-layer DNN model	0.086	0.849	72.6
6-layer DNN model	0.264	0.762	79.0
8-layer ResNet-like model	<0.2	0.982	66.1
**Deeper-net deep learning models developed**			
**in this work**			
14-layer ResNet-like model	0.133	0.827	74.2
20-layer ResNet-like model	**0.412**	**0.681**	**82.3**
26-layer ResNet-like model	0.075	0.854	77.4

a *The performance of the established tools, and the shallow-net and deeper-net deep learning models in the prediction of experimental logS values of 62 recently-published novel compounds. The best performance values are in bold font*.

b *Percent of predicted logS value within 10-fold of experimental value*.

### Comparison With Human Experts in Coarse-Grained Classification of the Solubility Categories of the Literature-Reported Novel Compounds

Four human experts in medicinal chemistry were selected from the China Pharmaceutical University using the criterion of a recent machine vs. human comparative solubility prediction study (9), i.e., a human expert is someone with medicinal chemistry expertise working or studying in a university. These four experts include one assistant professor and three PhD students. They were tasked to conduct coarse-grained classification of the aqueous solubility of the 62 novel compounds at room temperature into one of the following categories: practically insoluble or insoluble (<0.1 g/L), slightly soluble (0.1~10 g/L), soluble (10~100 g/L), and freely soluble (>100 g/L). The classification performance of these four experts together with those of the established tools, and shallow and deeper-net models are in Table 2. All tools and models achieved high classification accuracies of 79.0–91.9%, which significantly outperformed the human experts (6.5–74.2%). These indicated the more superior capability of both established tools and deep learning models over human experts in coarse-grained classification of the solubility categories on novel compounds. However, no definite conclusion could be deduced on which was better between the established tools and the deep learning models. No improving trend was found with the increasing of the deep learning models' depth. It seemed that the coarse-grained classification method was not discriminative enough to differentiate the capabilities of the established tools and deep learning models as revealed by the more quantitatively-precise evaluations of R^2^ and RMSE values.

**Table 2:** Performance on the solubility category prediction^a^.

**Human expert or established tool**	**Percent of 62 compounds with correct classification (%)**	**Deep learning model**	**Percent of 62 compounds with correct classification (%)**
Expert 1	6.5	4-layer DNN model	79.0
Expert 2	8.1	1-layer DNN model	79.0
Expert 3	11.3	6-layer DNN model	82.3
Expert 4	74.2	8-layer ResNet-like model	80.7
MOE V2016.0802	91.9	14-layer ResNet-like model	87.1
QikProp 2018-4 QP18	85.5	20-layer ResNet-like model	85.5
QikProp 2018-4 CIQP18	87.1	26-layer ResNet-like model	83.9
AlogGPS V2.1	82.3		

a *The performance of human experts, the established tools, and the shallow-net and deeper-net deep learning models in the prediction of solubility category of 62 recently-published novel compounds. The solubility categories are practically insoluble or insoluble (<0.1 g/L), slightly soluble (0.1–10 g/L), soluble (10–100 g/L), and freely soluble (>100 g/L)*.

### Solubility Prediction of a Series of Novel Compounds From a Real Anti-cancer Drug Discovery Project

**Figure 3:**
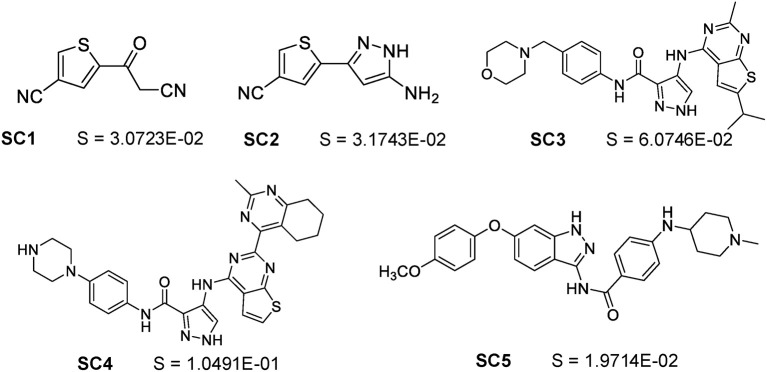
The molecular structures and experimental solubility S values (in mg/mL) of the five synthetic novel compounds for a drug discovery project with solubility values measured for the first time by this work.

A series of 17 novel compounds were synthesized by using the method described in Supplementary Method 1 and the published literatures (36–39) for discovering FLT3 inhibitors. These compounds are structurally novel based on SciFinder search. They are difficult for solubility estimation based on our surveys with medicinal chemistry experts. The solubility values of these 17 compounds (Supplementary Figure 4) were experimentally measured using the method described in the Methods section. We were unable to determine the exact solubility values for 12 compounds because they are insoluble below 1.0000E-2 mg/mL in neutral water. Hence, only the remaining five compounds (Figure 3) with exact experimental solubility values were used for testing our deeper-net models and other models. Partly because of the novelty and low number of compounds, the R^2^ values of all models are well below statistically meaningful values. Hence only the RMSE values and the percent of predicted logS values within 10-fold of experimental value were used for performance evaluation (Table 3). Judged by the RMSE values, the deeper-net models substantially outperformed all other models, with the 26-layer model as the best one in spite of minor level of overfitting. This further indicated the advantage of going deeper for improved solubility prediction. Judged by the percent of predicted logS values within 10-fold of experimental value, the majority of the models (including 14- and 20-layer deeper-net models) achieved equally good performances (60%) with the 26-layer model as the best one (80%). This again showed that both the established tools and deep learning models are useful for rough estimation of the solubility values of novel compounds.

**Table 3:** Performance on the logS prediction of 5 novel compounds^a^.

**Model**	**RMSE**	**PCT-10fold^b^ (%)**
**Established tools**		
MOE V2016.0802	2.293	<20
QikProp 2018-4 QP18	2.717	20
QikProp 2018-4 CIQP18	2.308	20
AlogGPS V2.1	1.073	60
**Shallow-net deep learning model of a typically-employed**		
**architecture for solubility prediction**		
4-layer DNN model	1.325	60
**Shallow-net deep learning models developed in**		
**this work**		
1-layer DNN model	1.502	60
6-layer DNN model	1.494	40
8-layer ResNet-like model	1.646	60
**Deeper-net deep learning models developed in**		
**this work**		
14-layer ResNet-like model	0.982	60
20-layer ResNet-like model	0.811	60
26-layer ResNet-like model	**0.689**	**80**

a *The performance of the established tools, and the shallow-net and deeper-net deep learning models in the prediction of experimental logS values of 5 novel compounds (quantitative values measured in this work). The best performance value is in bold font*.

b *Percent of predicted logS value within 10-fold of experimental value*.

## Discussions

Like successful applications of deep learning methods in other fields (19–21), the superior learning capability of deeper-net models may be exploited to improve solubility prediction of novel compounds, including those compounds considered by medicinal chemistry experts as difficult for solubility estimations. To better explore the learning capability of deeper-net architectures, the molecular representations of the compounds may be selected for conforming to these architectures. Specifically, the superior local-feature learning capability of the CNN architectures may be better exploited by using the substructure-encoded molecular fingerprints for representing compounds. Our studies consistently scored the substantially better solubility prediction performances of the deeper-net deep learning models on novel compounds than the established tools and shallow-net models. Nonetheless, the prediction performance of the deeper-net models on novel compounds is affected by the limited number of 9,943 compounds for training these models. Solubility prediction capability of the deeper-net methods may be further enhanced with the expanded experimental solubility data and by means of algorithm development. Our novel approach may find broader applications in the development of high-performance deep learning models for the prediction of various pharmacodynamic, pharmacokinetic, and toxicological properties.

## Data Availability Statement

All datasets generated for this study are included in the article/Supplementary Material.

## Author Contributions

YC and HZ conceived and designed this research, contributed to the preparation, and write-up of this manuscript. HZ designed and conducted computation. QC processed data, built models, predicted results, analyzed results data, and participated in this manuscript writing. SL synthesized compounds and experimented to test the aqueous solubility values of these compounds, and participated in this manuscript writing. BN collected and processed information on 62 recently-published novel compounds and assisted in optimizing the models. XZ predicted the aqueous solubility values of novel compounds using commercial software. YT assisted in the design of this study.

### Conflict of Interest

The authors declare that the research was conducted in the absence of any commercial or financial relationships that could be construed as a potential conflict of interest.

## Footnotes

^1^http://www.rdkit.org/

^2^http://www.chemcomp.com/index.htm

^3^https://www.schrodinger.com/QikProp
