# Supplementary material for: Improved Prediction of Aqueous Solubility of Novel Compounds by Going Deeper With Deep Learning
Source: Front Oncol. 2020 Feb 11;10:121. doi: 10.3389/fonc.2020.00121 (PMC7026387; doi:10.3389/fonc.2020.00121)
Supplement: Supplementary file 1 [file Data_Sheet_1.ZIP › Supplementary Material/Supplementary Material.pdf]

frontiers

Supplementary Material

1 Supplementary Figures and Tables

1.1 Supplementary Figures

Supplementary Figure S1. 62 recently-published novel compounds with experimental solubility S values

Novel Compound ID: NC1 (Theppawong et al., 2018)

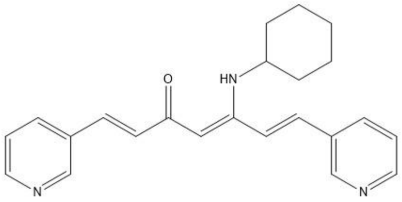

SMILES: O=C(C=C(NC1CCCCC1)C=Cc1cccnc1)C=Cc1cccnc1

Experimental S value: 11.3 μM (by shake flask method and colorimetry)

Novel Compound ID: NC2 (Wilson et al., 2017)

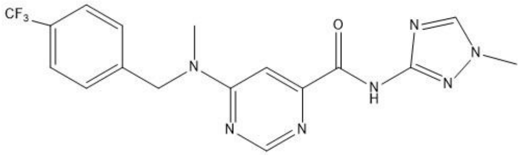

SMILES: Cn1cnc(n1)NC(=O)c1ncnc(c1)N(Cc1ccc(cc1)C(F)(F)F)C

Experimental S value: 173 μM (NA) [a]

Novel Compound ID: NC3 (Thompson et al., 2018)

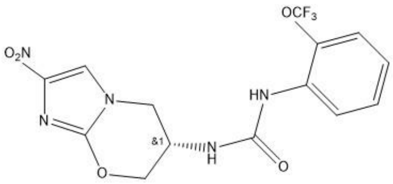

SMILES: O=C(NC1=CC=CC=C1OC(F)(F)F)N[C@@H]2COC3=NC([N+]([O-])=O)=CN3C2

Experimental S value: 22 μg/mL (by HPLC)

Novel Compound ID: NC4 (Hamdy et al., 2017)

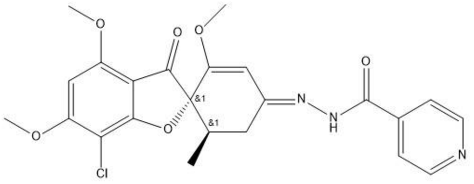

SMILES: COC1=CC(=NNC(=O)c2ccncc2)CC(C21Oc1c(C2=O)c(OC)cc(c1Cl)OC)C

Experimental S value: 12.53 µg/mL (NA)

Novel Compound ID: NC5 (Hamdy et al., 2017)

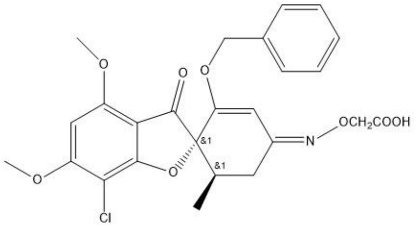

SMILES: COc1cc(OC)c(c2c1C(=O)C1(O2)C(C)CC(=NOCC(=O)O)C=C1OCc1ccccc1)Cl

Experimental S value: 27.94 µg/mL (NA)

Novel Compound ID: NC6 (Theppawong et al., 2018)

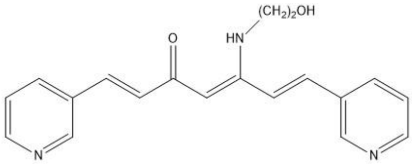

SMILES: OCCNC(=CC(=O)C=Cc1cccnc1)C=Cc1cccnc1

Experimental S value: 4350.8 µM (by shake flask method and colorimetry)

Novel Compound ID: NC7 (Thompson et al., 2018)

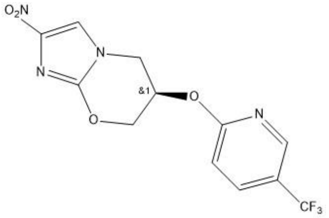

SMILES: O=[N+](C1=CN2C(OC[C@H](OC3=CC=C(C=N3)C(F)(F)F)C2)=N1)[O-]

Experimental S value: 110 µg/mL (by HPLC)

Novel Compound ID: NC8 (Thompson et al., 2018)

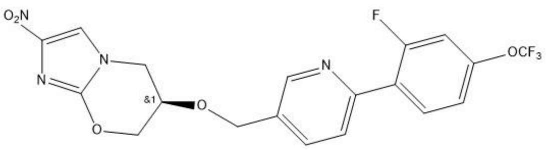

SMILES:
FC1=CC(OC(F)(F)F)=CC=C1C(N=C2)=CC=C2CO[C@H]3COC4=NC([N+]([O-])=O)=CN4C3

Experimental S value: 2.9 μg/mL (by HPLC)

Novel Compound ID: NC9 (Thompson et al., 2018)

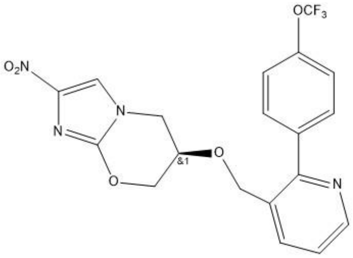

SMILES:
O=[N+](C1=CN2C(OC[C@H](OCC3=CC=CN=C3C4=CC=C(C=C4)OC(F)(F)F)C2)=N1)[O-]

Experimental S value: 0.51 μg/mL (by HPLC)

Novel Compound ID: NC10 (Ortiz et al., 2017)

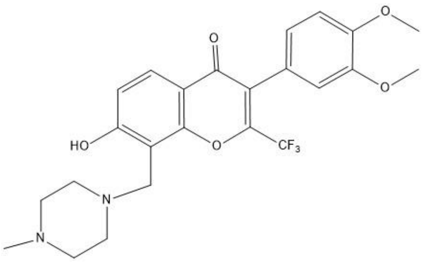

SMILES: COc1cc(ccc1OC)c1c(=O)c2ccc(c(c2oc1C(F)(F)F)CN1CCN(CC1)C)O

Experimental S value: 67 μM (NA)

Novel Compound ID: NC11 (Ortiz et al., 2017)

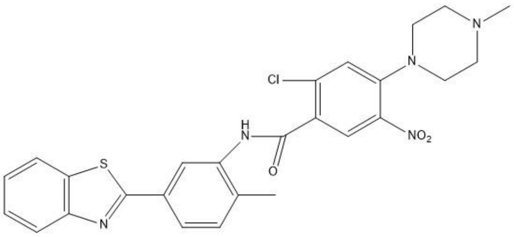

SMILES: CN1CCN(CC1)c1cc(Cl)c(cc1[N+](=O)[O-])C(=O)Nc1cc(ccc1C)c1nc2c(s1)cccc2

Experimental S value: 0.3 μM (NA)

Novel Compound ID: NC12 (Wilson et al., 2017)

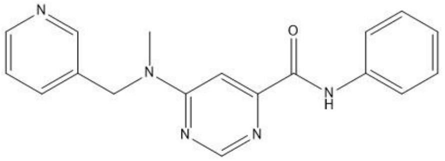

SMILES: CN(c1ncnc(c1)C(=O)Nc1ccccc1)Cc1cccnc1

Experimental S value: 150 µM (NA)

Novel Compound ID: NC13 (Wilson et al., 2017)

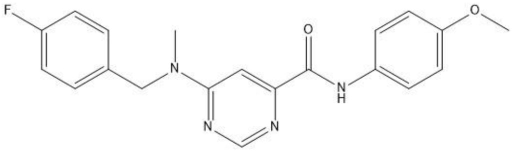

SMILES: COc1ccc(cc1)NC(=O)c1ncnc(c1)N(Cc1ccc(cc1)F)C

Experimental S value: 62 µM (NA)

Novel Compound ID: NC14 (Wilson et al., 2017)

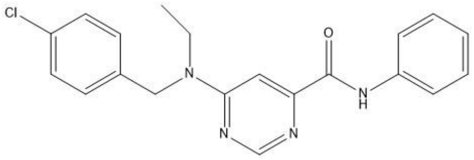

SMILES: CCN(c1ncnc(c1)C(=O)Nc1ccccc1)Cc1ccc(cc1)Cl

Experimental S value: 5 µM (NA)

Novel Compound ID: NC15 (Wilson et al., 2017)

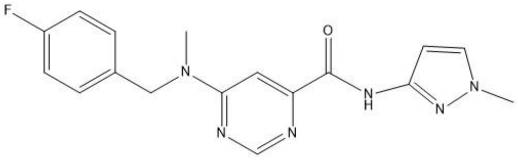

SMILES: FC1=CC=C(CN(C)C2=NC=NC(C(NC3=NN(C)C=C3)=O)=C2)C=C1

Experimental S value: 90 µM (NA)

Novel Compound ID: NC16 (Theppawong et al., 2018)

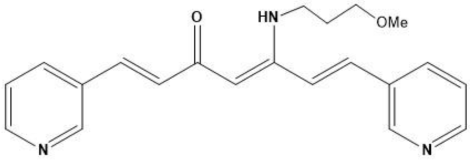

SMILES: O=C(/C=C(NCCCOC)/C=C/C1=CN=CC=C1)/C=C/C2=CC=CN=C2

Experimental S value: 897.4 µM (by shake flask method and colorimetry)

Novel Compound ID: NC17 (Theppawong et al., 2018)

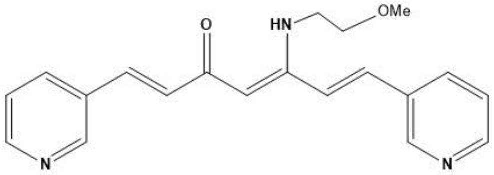

SMILES: O=C(/C=C(NCCOC)/C=C/C1=CN=CC=C1)/C=C/C2=CC=CN=C2

Experimental S value: 895 µM (by shake flask method and colorimetry)

Novel Compound ID: NC18 (Thompson et al., 2018)

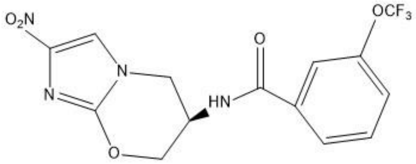

SMILES: O=C(C1=CC(OC(F)(F)F)=CC=C1)N[C@H]2COC3=NC([N+]([O-])=O)=CN3C2

Experimental S value: 85 µg/mL (by HPLC)

Novel Compound ID: NC19 (Thompson et al., 2018)

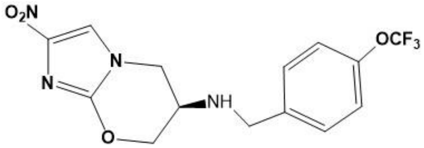

SMILES: O=[N+](C1=CN2C(OC[C@H](NCC3=CC=C(C=C3)OC(F)(F)F)C2)=N1)[O-]

Experimental S value: 84 µg/mL (by HPLC)

Novel Compound ID: NC20 (Thompson et al., 2018)

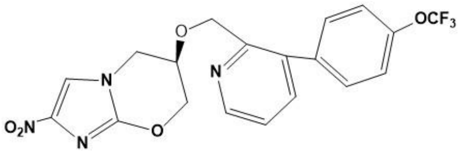

SMILES:
O=[N+](C1=CN2C(OC[C@H](OCC3=NC=CC=C3C4=CC=C(C=C4)OC(F)(F)F)C2)=N1)[O-]

Experimental S value: 78 µg/mL (by HPLC)

Novel Compound ID: NC21 (Wilson et al., 2017)

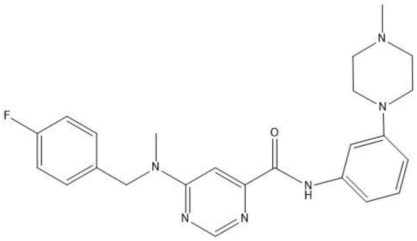

SMILES: FC1=CC=C(CN(C)C2=NC=NC(C(NC3=CC=CC(N4CCN(C)CC4)=C3)=O)=C2)C=C1

Experimental S value: 74 μM (NA)

Novel Compound ID: NC22 (Thompson et al., 2018)

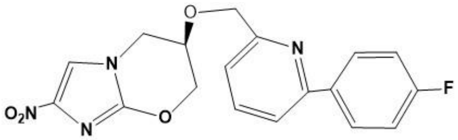

SMILES: FC1=CC=C(C2=NC(CO[C@H]3COC4=NC([N+]([O-])=O)=CN4C3)=CC=C2)C=C1

Experimental S value: 6 μg/mL (by HPLC)

Novel Compound ID: NC23 (Thompson et al., 2018)

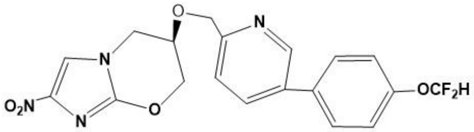

SMILES:
O=[N+](C1=CN2C(OC[C@H](OCC3=CC=C(C4=CC=C(C=C4)OC(F)F)C=N3)C2)=N1)[O-]

Experimental S value: 5.7 μg/mL (by HPLC)

Novel Compound ID: NC24 (Wilson et al., 2017)

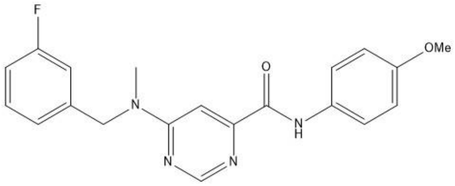

SMILES: CN(C1=NC=NC(C(NC2=CC=C(OC)C=C2)=O)=C1)CC3=CC=CC(F)=C3

Experimental S value: 5 μM (NA)

Novel Compound ID: NC25 (Wilson et al., 2017)

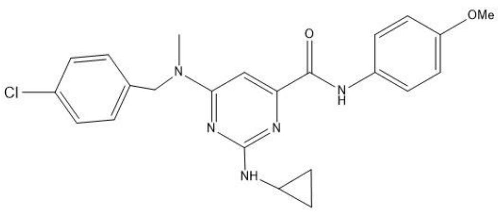

SMILES: O=C(NC1=CC=C(OC)C=C1)C2=CC(N(C)CC3=CC=C(Cl)C=C3)=NC(NC4CC4)=N2

Experimental S value: 5 μM (NA)

Novel Compound ID: NC26 (Wilson et al., 2017)

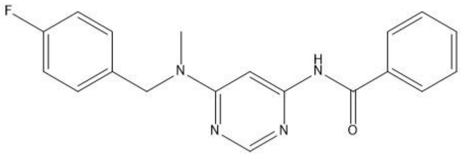

SMILES: FC1=CC=C(CN(C)C2=NC=NC(NC(C3=CC=CC=C3)=O)=C2)C=C1

Experimental S value: 5 μM (NA)

Novel Compound ID: NC27 (Wilson et al., 2017)

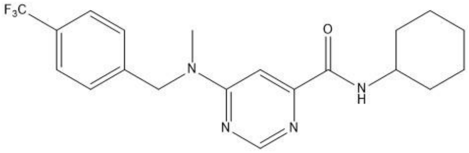

SMILES: CN(C1=NC=NC(C(NC2CCCCC2)=O)=C1)CC(C=C3)=CC=C3C(F)(F)F

Experimental S value: 5 μM (NA)

Novel Compound ID: NC28 (Wilson et al., 2017)

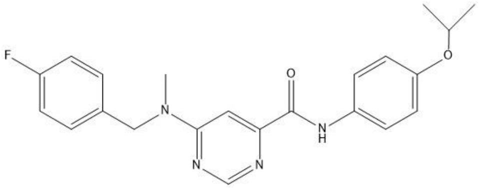

SMILES: FC1=CC=C(CN(C)C2=NC=NC(C(NC3=CC=C(OC(C)C)C=C3)=O)=C2)C=C1

Experimental S value: 5 μM (NA)

Novel Compound ID: NC29 (Thompson et al., 2018)

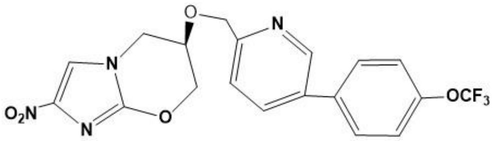

SMILES:
O=[N+](C1=CN2C(OC[C@H](OCC3=CC=C(C4=CC=C(C=C4)OC(F)(F)F)C=N3)C2)=N1)[O-]

Experimental S value: 3 μg/mL (by HPLC)

Novel Compound ID: NC30 (Thompson et al., 2018)

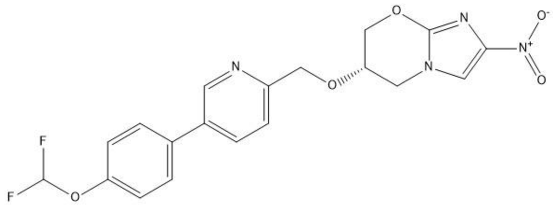

SMILES:
O=[N+](C1=CN2C(OC[C@@H](OCC3=CC=C(C4=CC=C(C=C4)OC(F)F)C=N3)C2)=N1)[O-]

Experimental S value: 4 μg/mL (by HPLC)

Novel Compound ID: NC31 (Thompson et al., 2018)

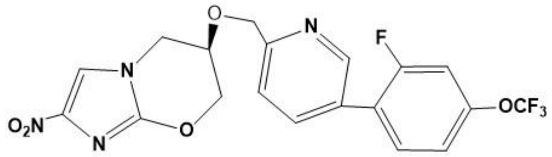

SMILES:
FC1=CC(OC(F)(F)F)=CC=C1C2=CC=C(CO[C@H]3COC4=NC([N+]([O-])=O)=CN4C3)N=C2

Experimental S value: 3.4 μg/mL (by HPLC)

Novel Compound ID: NC32 (Wilson et al., 2017)

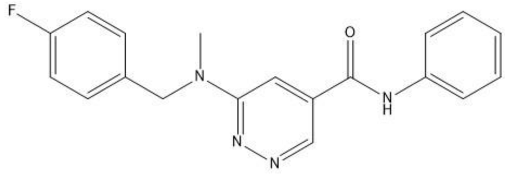

SMILES: CN(C1=NN=CC(C(NC2=CC=CC=C2)=O)=C1)CC3=CC=C(F)C=C3

Experimental S value: 40 μM (NA)

Novel Compound ID: NC33 (Thompson et al., 2018)

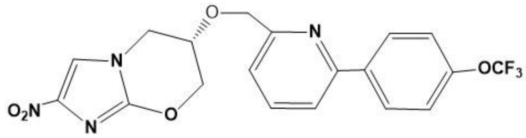

SMILES:
O=[N+](C1=CN2C(OC[C@@H](OCC3=CC=CC(C4=CC=C(C=C4)OC(F)(F)F)=N3)C2)=N1)[O-]

Experimental S value: 3 μg/mL (by HPLC)

Novel Compound ID: NC34 (Theppawong et al., 2018)

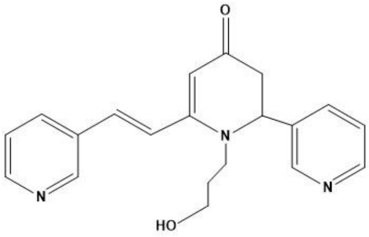

SMILES: O=C1CC(C2=CN=CC=C2)N(CCCO)C(/C=C/C3=CC=CN=C3)=C1

Experimental S value: 2462.6 μM (by shake flask method and colorimetry)

Novel Compound ID: NC35 (Thompson et al., 2018)

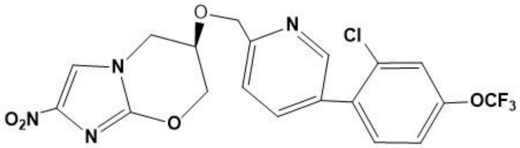

SMILES:
ClC1=CC(OC(F)(F)F)=CC=C1C2=CC=C(CO[C@H]3COC4=NC([N+]([O-])=O)=CN4C3)N=C2

Experimental S value: 2.9 μg/mL (by HPLC)

Novel Compound ID: NC36 (Thompson et al., 2018)

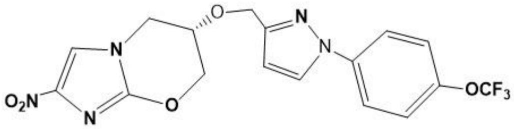

SMILES:
O=[N+](C1=CN2C(OC[C@@H](OCC(C=C3)=NN3C4=CC=C(C=C4)OC(F)(F)F)C2)=N1)[O-]

Experimental S value: 2.7 μg/mL (by HPLC)

Novel Compound ID: NC37 (Thompson et al., 2018)

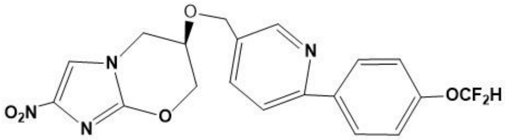

SMILES:
O=[N+](C1=CN2C(OC[C@H](OCC3=CC=C(C4=CC=C(C=C4)OC(F)F)N=C3)C2)=N1)[O-]

Experimental S value: 2.1 μg/mL (by HPLC)

Novel Compound ID: NC38 (Wilson et al., 2017)

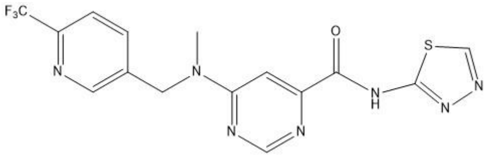

SMILES: CN(C1=NC=NC(C(NC2=NN=CS2)=O)=C1)CC(C=C3)=CN=C3C(F)(F)F

Experimental S value: 191 µM (NA)

Novel Compound ID: NC39 (Thompson et al., 2018)

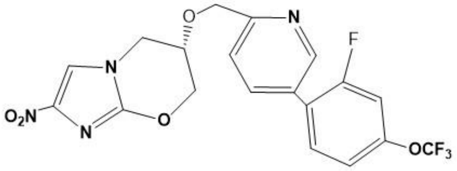

SMILES:
FC1=CC(OC(F)(F)F)=CC=C1C2=CC=C(CO[C@@H]3COC4=NC([N+]([O-])=O)=CN4C3)N=C2

Experimental S value: 1.4 µg/mL (by HPLC)

Novel Compound ID: NC40 (Wilson et al., 2017)

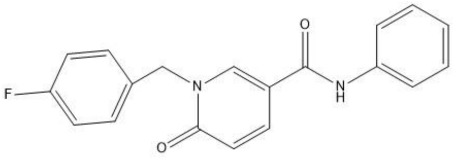

SMILES: O=C(NC1=CC=CC=C1)C2=CN(C(C=C2)=O)CC3=CC=C(F)C=C3

Experimental S value: 170 µM (NA)

Novel Compound ID: NC41 (Thompson et al., 2018)

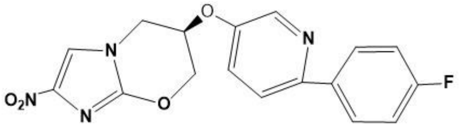

SMILES: FC1=CC=C(C2=CC=C(O[C@H]3COC4=NC([N+]([O-])=O)=CN4C3)C=N2)C=C1

Experimental S value: 1.2 µg/mL (by HPLC)

Novel Compound ID: NC42 (Wilson et al., 2017)

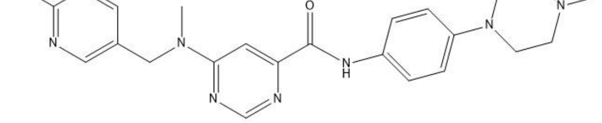

SMILES:
CN(C1=NC=NC(C(NC2=CC=C(N3CCN(C)CC3)C=C2)=O)=C1)CC(C=C4)=CN=C4C(F)(F)F

Experimental S value: 165 μM (NA)

Novel Compound ID: NC43 (Hamdy et al., 2017)

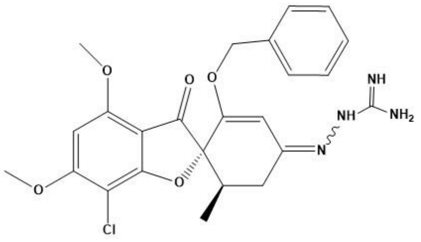

SMILES: COC1=C2C(O[C@]3([C@H](C)C/C(C=C3OCC4=CC=CC=C4)=N/NC(N)=N)C2=O)=C(Cl)C(OC)=C1

Experimental S value: 16.58 μg/mL (NA)

Novel Compound ID: NC44 (Wilson et al., 2017)

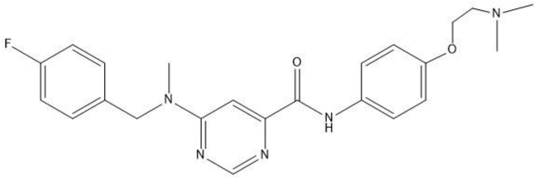

SMILES: FC1=CC=C(CN(C)C2=NC=NC(C(NC3=CC=C(OCCN(C)C)C=C3)=O)=C2)C=C1

Experimental S value: 155 μM (NA)

Novel Compound ID: NC45 (Wilson et al., 2017)

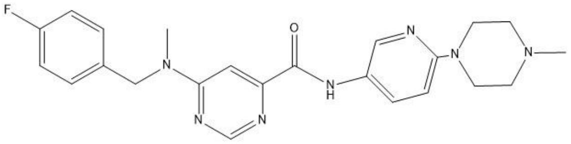

SMILES: FC1=CC=C(CN(C)C2=NC=NC(C(NC3=CC=C(N4CCN(C)CC4)N=C3)=O)=C2)C=C1

Experimental S value: 150 μM (NA)

Novel Compound ID: NC46 (Thompson et al., 2018)

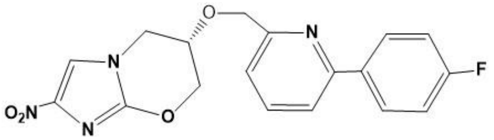

SMILES: FC1=CC=C(C2=NC(CO[C@@H]3COC4=NC([N+]([O-])=O)=CN4C3)=CC=C2)C=C1

Experimental S value: 15 μg/mL (by HPLC)

Novel Compound ID: NC47 (Theppawong et al., 2018)

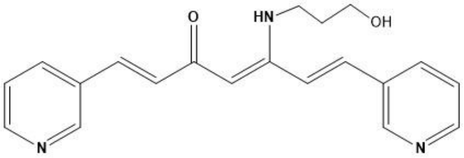

SMILES: O=C(/C=C(NCCCO)/C=C/C1=CN=CC=C1)/C=C/C2=CC=CN=C2

Experimental S value: 1472.8 μM (by shake flask method and colorimetry)

Novel Compound ID: NC48 (Hamdy et al., 2017)

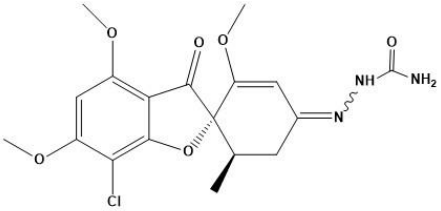

SMILES: COC1=C2C(O[C@]3([C@H](C)C/C(C=C3OC)=N/NC(N)=O)C2=O)=C(Cl)C(OC)=C1

Experimental S value: 14.51 μg/mL (NA)

Novel Compound ID: NC49 (Thompson et al., 2018)

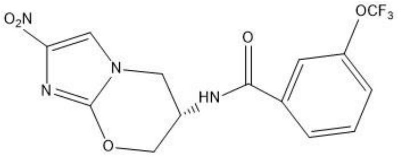

SMILES: O=C(C1=CC(OC(F)(F)F)=CC=C1)N[C@@H]2COC3=NC([N+]([O-])=O)=CN3C2

Experimental S value: 132 μg/mL (by HPLC)

Novel Compound ID: NC50 (Hamdy et al., 2017)

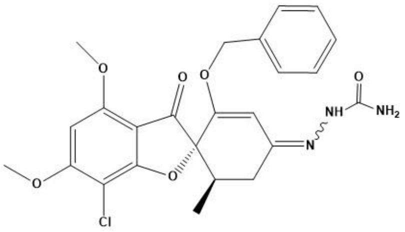

SMILES:
COC1=C2C(O[C@]3([C@H](C)C/C(C=C3OCC4=CC=CC=C4)=N/NC(N)=O)C2=O)=C(Cl)C(OC)
=C1

Experimental S value: 13.78 μg/mL (NA)

Novel Compound ID: NC51 (Wilson et al., 2017)

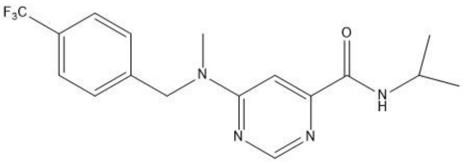

SMILES: CN(C1=NC=NC(C(NC(C)C)=O)=C1)CC(C=C2)=CC=C2C(F)(F)F

Experimental S value: 13 μM (NA)

Novel Compound ID: NC52 (Theppawong et al., 2018)

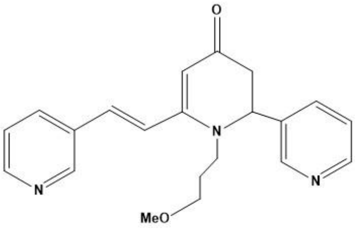

SMILES: O=C1CC(C2=CN=CC=C2)N(CCCOC)C(/C=C/C3=CC=CN=C3)=C1

Experimental S value: 1283.1 μM (by shake flask method and colorimetry)

Novel Compound ID: NC53 (Wilson et al., 2017)

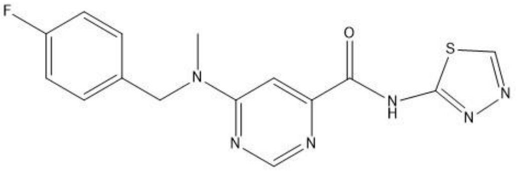

SMILES: FC1=CC=C(CN(C)C2=NC=NC(C(NC3=NN=CS3)=O)=C2)C=C1

Experimental S value: 128 μM (NA)

Novel Compound ID: NC54 (Thompson et al., 2018)

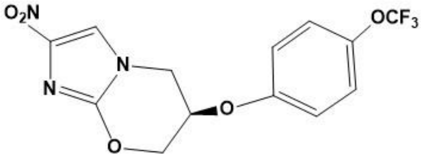

SMILES: O=[N+](C1=CN2C(OC[C@H](OC3=CC=C(C=C3)OC(F)(F)F)C2)=N1)[O-]

Experimental S value: 12 μg/mL (by HPLC)

Novel Compound ID: NC55 (Thompson et al., 2018)

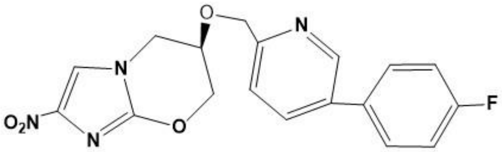

SMILES: FC1=CC=C(C2=CC=C(CO[C@H]3COC4=NC([N+]([O-])=O)=CN4C3)N=C2)C=C1

Experimental S value: 11 µg/mL (by HPLC)

Novel Compound ID: NC56 (Wilson et al., 2017)

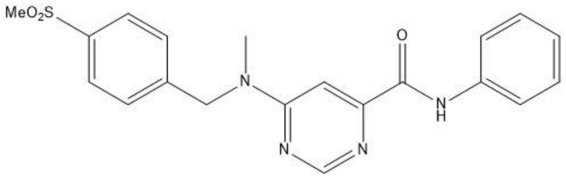

SMILES: CN(C1=NC=NC(C(NC2=CC=CC=C2)=O)=C1)CC3=CC=C(S(=O)(C)=O)C=C3

Experimental S value: 10 µM (NA)

Novel Compound ID: NC57 (Thompson et al., 2018)

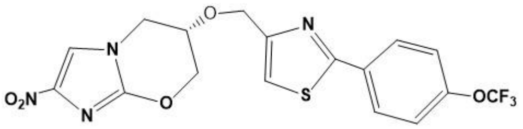

SMILES:
O=[N+](C1=CN2C(OC[C@@H](OCC3=CSC(C4=CC=C(C=C4)OC(F)(F)F)=N3)C2)=N1)[O-]

Experimental S value: 1.6 µg/mL (by HPLC)

Novel Compound ID: NC58 (Thompson et al., 2018)

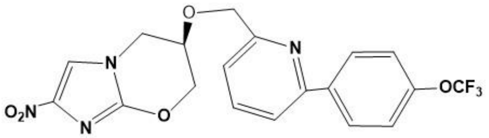

SMILES:
O=[N+](C1=CN2C(OC[C@H](OCC3=CC=CC(C4=CC=C(C=C4)OC(F)(F)F)=N3)C2)=N1)[O-]

Experimental S value: 1.5 µg/mL (by HPLC)

Novel Compound ID: NC59 (Hamdy et al., 2017)

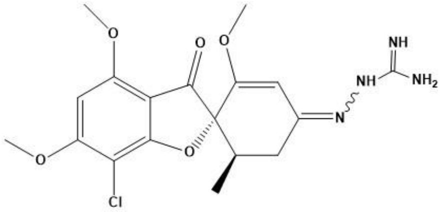

SMILES: COC1=C2C(O[C@]3([C@H](C)C/C(C=C3OC)=N/NC(N)=N)C2=O)=C(Cl)C(OC)=C1

Experimental S value: 18.32 µg/mL (NA)

Novel Compound ID: NC60 (Thompson et al., 2018)

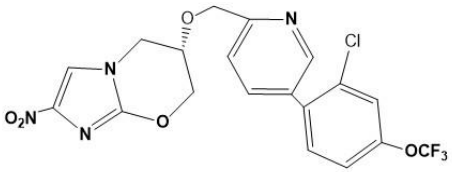

SMILES:
ClC1=CC(OC(F)(F)F)=CC=C1C2=CC=C(CO[C@@H]3COC4=NC([N+]([O-])=O)=CN4C3)N=C2

Experimental S value: 1.4 µg/mL (by HPLC)

Novel Compound ID: NC61 (Theppawong et al., 2018)

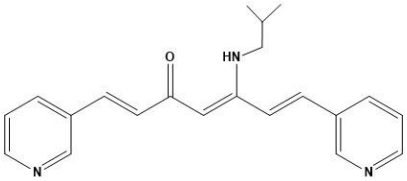

SMILES: O=C(/C=C(NCC(C)C)/C=C/C1=CN=CC=C1)/C=C/C2=CC=CN=C2

Experimental S value: 168.4 µM (by shake flask method and colorimetry)

Novel Compound ID: NC62 (Thompson et al., 2018)

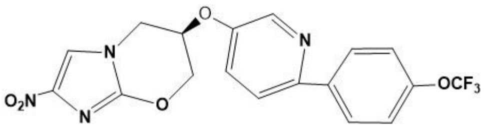

SMILES:
O=[N+](C1=CN2C(OC[C@H](OC3=CC=C(C4=CC=C(C=C4)OC(F)(F)F)N=C3)C2)=N1)[O-]

Experimental S value: 0.36 µg/mL (by HPLC)

[a]NA: Not Available.

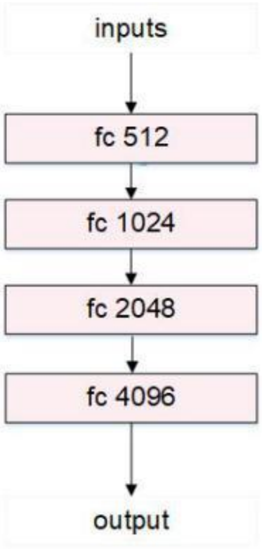

Supplementary Figure S2. The architecture of the 4-layer DNN model

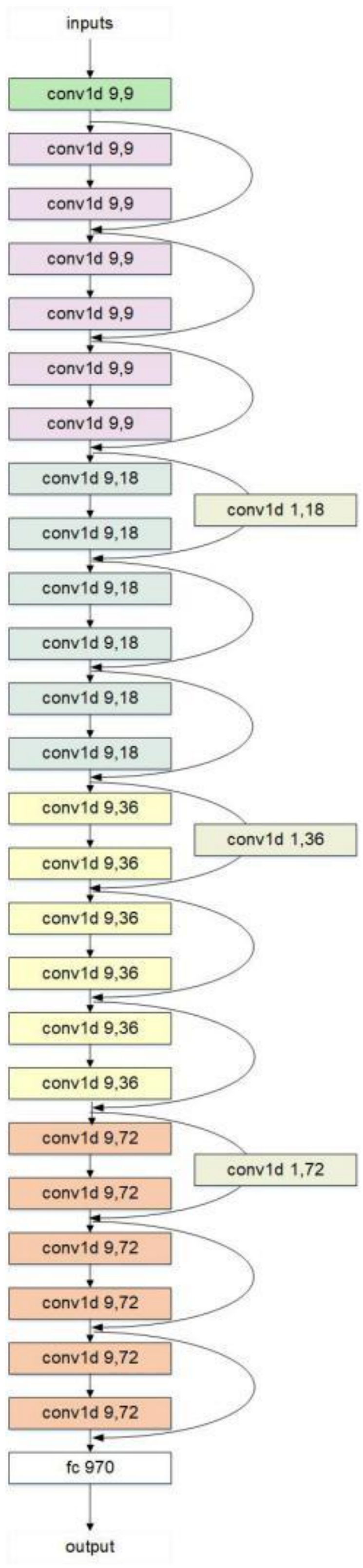

Supplementary Figure S3. The architecture of the 26-layer CNN ResNet-like deep learning model

Supplementary Figure S4. 17 synthetic novel compounds with experimental solubility S values in mg/mL

Synthetic Compound ID: SC1

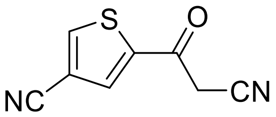

SMILES: O=C(CC#N)C1=CC(C#N)=CS1

Experimental S value: 3.0723E-02

Synthetic Compound ID: SC2

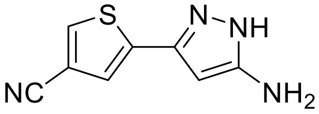

SMILES: NC1=CC(C2=CC(C#N)=CS2)=NN1

Experimental S value: 3.1743E-02

Synthetic Compound ID: SC3

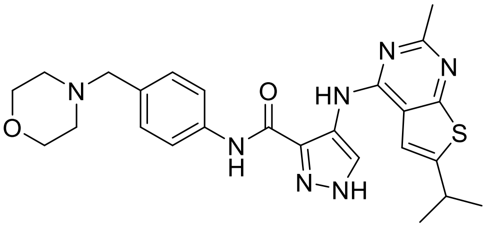

SMILES:
O=C(C1=NNC=C1NC2=C(C=C(C(C)C)S3)C3=NC(C)=N2)NC(C=C4)=CC=C4CN5CCOCC5

Experimental S value: 6.0746E-02

Synthetic Compound ID: SC4

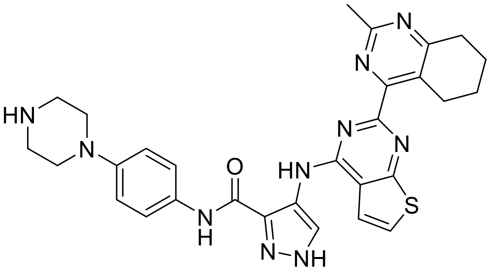

SMILES:
O=C(C1=NNC=C1NC2=C3C(SC=C3)=NC(C4=C(CCCC5)C5=NC(C)=N4)=N2)NC(C=C6)=CC=C6N7CCNCC7

Experimental S value: 1.0491E-01

Synthetic Compound ID: SC5

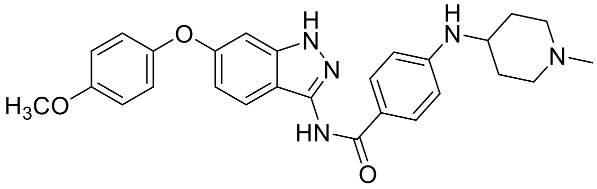

SMILES: O=C(NC1=NNC2=CC(OC3=CC=C(OC)C=C3)=CC=C21)C4=CC=C(NC5CCN(C)CC5)C=C4

Experimental S value: 1.9714E-02

Synthetic Compound ID: SC6

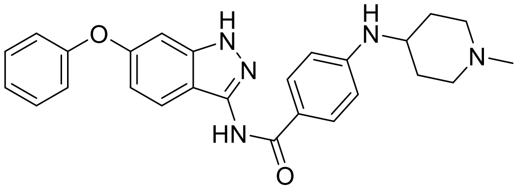

SMILES: O=C(NC1=NNC2=CC(OC3=CC=CC=C3)=CC=C21)C4=CC=C(NC5CCN(C)CC5)C=C4

Experimental S value: <1.0000E-02

Synthetic Compound ID: SC7

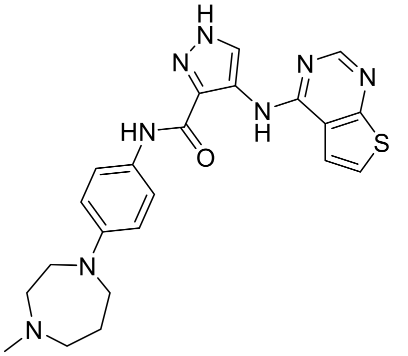

SMILES: O=C(NC1=CC=C(N2CCN(C)CCC2)C=C1)C3=NNC=C3NC4=NC=NC5=C4C=CS5

Experimental S value: <1.0000E-02

Synthetic Compound ID: SC8

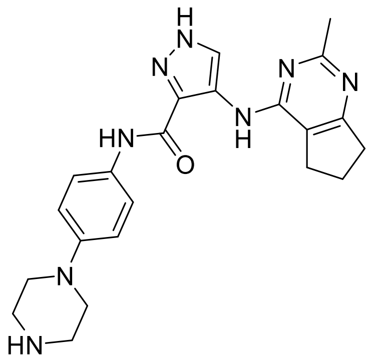

SMILES: O=C(NC1=CC=C(N2CCNCC2)C=C1)C3=NNC=C3NC4=NC(C)=NC5=C4CCC5

Experimental S value: <1.0000E-02

Synthetic Compound ID: SC9

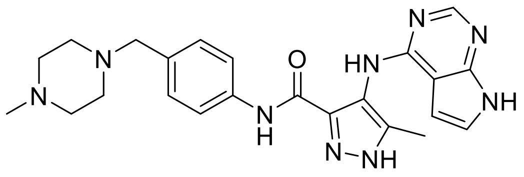

SMILES: O=C(C1=NNC(C)=C1NC2=C(C=CN3)C3=NC=N2)NC(C=C4)=CC=C4CN5CCN(C)CC5

Experimental S value: <1.0000E-02

Synthetic Compound ID: SC10

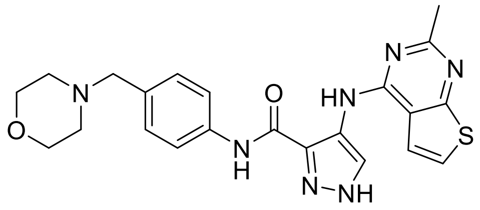

SMILES: O=C(C1=NNC=C1NC2=C(C=CS3)C3=NC(C)=N2)NC(C=C4)=CC=C4CN5CCOCC5

Experimental S value: <1.0000E-02

Synthetic Compound ID: SC11

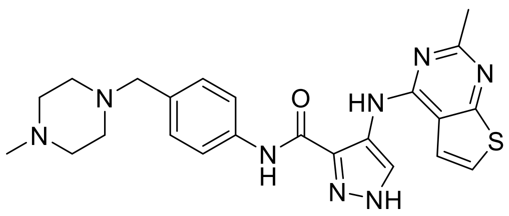

SMILES: O=C(C1=NNC=C1NC2=C(C=CS3)C3=NC(C)=N2)NC(C=C4)=CC=C4CN5CCN(C)CC5

Experimental S value: <1.0000E-02

Synthetic Compound ID: SC12

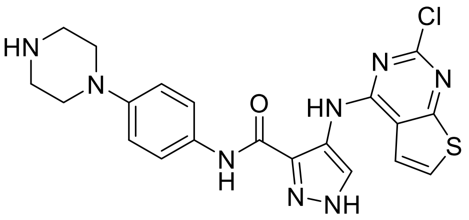

SMILES: O=C(C1=NNC=C1NC2=C(C=CS3)C3=NC(Cl)=N2)NC4=CC=C(N5CCNCC5)C=C4

Experimental S value: <1.0000E-02

Synthetic Compound ID: SC13

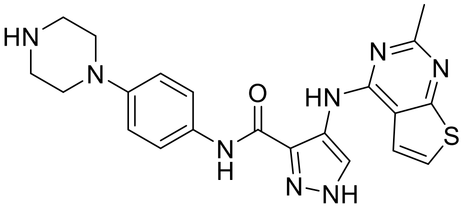

SMILES: O=C(C1=NNC=C1NC2=C(C=CS3)C3=NC(C)=N2)NC4=CC=C(N5CCNCC5)C=C4

Experimental S value: <1.0000E-02

Synthetic Compound ID: SC14

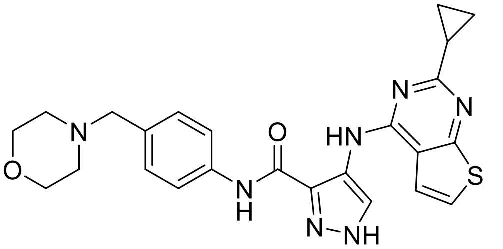

SMILES:
O=C(C1=NNC=C1NC2=C(C=CS3)C3=NC(C4CC4)=N2)NC5=CC=C(CN6CCOCC6)C=C5

Experimental S value: <1.0000E-02

Synthetic Compound ID: SC15

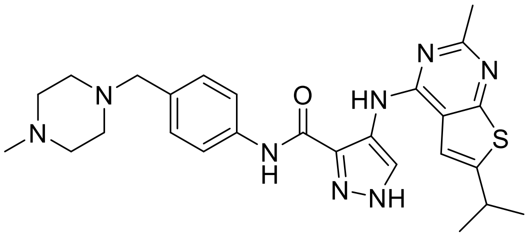

SMILES:
O=C(C1=NNC=C1NC2=C(C=C(C(C)C)S3)C3=NC(C)=N2)NC(C=C4)=CC=C4CN5CCN(C)CC5

Experimental S value: <1.0000E-02

Synthetic Compound ID: SC16

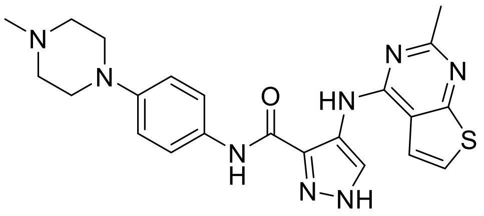

SMILES: O=C(C1=NNC=C1NC2=C(C=CS3)C3=NC(C)=N2)NC(C=C4)=CC=C4N5CCN(C)CC5

Experimental S value: <1.0000E-02

Synthetic Compound ID: SC17

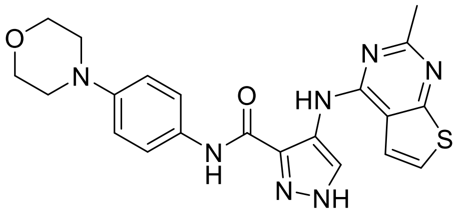

SMILES: O=C(C1=NNC=C1NC2=C(C=CS3)C3=NC(C)=N2)NC(C=C4)=CC=C4N5CCOCC5

Experimental S value: <1.0000E-02

1.2 Supplementary Tables

Supplementary Table S2. The basic physical properties of the training dataset

Distribution Properties	logP[a]	MW[b]	logS
min	-10.93	16.043	-18.22
max	20.85	1583.6	1.7
mean	2.40	292.52	-2.96
sd	2.30	138.78	2.21

[a]Octanol-water log P. [b]Molecular weight.

Supplementary Table S3. The scanned and optimized hyperparameters of the 20-layer deeper-net deep learning model. The optimized hyperparameters are in bold font.

**Hyperparameter**	**Optimal value**
Loss Function	**-R^2**
Size of convolution kernels	{ **9**, 49 }
Number of filters of CNN	{ **9**, 18 }
Strides	{ **1**, 2 }
Number of layers of CNN	**19**
Number of fully-connected hidden layers	**1**
Number of neurons of fully-connected layer	{ 256, **970** ,1280 }
Activation Function	**ReLU**
Optimizer	{ **Adam,** Nadam, PMSprop }
Learning Rate	{ 0.001, **0.0001** }
Weight initialization	{ **uniform**, he_normal }
L2 Weight Decay	{ **10^{-4}** , 10^{-5} }
Batch size	{ 30-60, **47** }
Epochs	{ 1500-**2000** }

Supplementary Table S4. The 10-fold cross validation performances of the 14-, 20- and 26-layer ResNet-like models

Supplementary Table S4. (A) The 10-fold cross validation performances of the 14-layer model

10-fold	1	2	3	4	5	6	7	8	9	10
R2	0.76	0.76	0.78	0.71	0.78	0.72	0.73	0.74	0.74	0.76
RMSE	1.109	1.078	1.041	1.141	0.988	1.144	1.134	1.098	1.052	1.033

Supplementary Table S4. (B) The 10-fold cross validation performances of the 20-layer model

10-fold	1	2	3	4	5	6	7	8	9	10
R2	0.77	0.76	0.79	0.75	0.77	0.74	0.77	0.76	0.75	0.77
RMSE	1.089	1.076	1.034	1.077	1.012	1.112	1.043	1.058	1.028	1.006

Supplementary Table S4. (C) The 10-fold cross validation performances of the 26-layer model

10-fold	1	2	3	4	5	6	7	8	9	10
R2	0.79	0.76	0.77	0.74	0.77	0.72	0.73	0.75	0.75	0.77
RMSE	1.037	1.064	1.066	1.095	1.029	1.151	1.136	1.077	1.033	1.015

Supplementary Table S5. The predicted logS values of the established tools, the shallow-net and deeper-net deep learning models with respect to experimental logS values of 62 recently-published novel compounds

**62-novel Compounds**	**1**[a]	**2**[b]	**3**[c]	**4**[d]	**5**[e]	**6**[f]	**7**[g]	**9**[h]	**10**[i]	**11**[j]	**12**[k]	**13**[l]
NC1	-4.947	-3.763	-5.233	-5.078	-5.32	-3.260	-2.998	-3.122	-2.249	-2.927	-4.061	-4.361
NC2	-3.762	-4.568	-5.260	-4.988	-4.17	-4.332	-3.790	-4.299	-4.355	-4.362	-4.089	-4.390
NC3	-4.246	-4.813	-4.633	-4.891	-3.83	-3.790	-2.850	-4.056	-3.315	-3.826	-3.493	-3.398
NC4	-4.576	-4.971	-5.937	-6.392	-4.68	-4.340	-3.834	-4.144	-4.175	-4.229	-4.109	-5.047
NC5	-4.254	-6.073	-6.468	-6.505	-5.34	-4.177	-4.189	-4.705	-4.190	-4.606	-3.741	-4.852
NC6	-2.361	-2.089	-3.483	-3.815	-4.11	-1.148	-1.336	-1.238	-2.454	-1.722	-2.249	-2.701
NC7	-3.477	-3.740	-3.924	-4.703	-3.59	-3.857	-3.495	-3.657	-2.666	-2.695	-3.467	-3.120
NC8	-5.195	-6.317	-5.678	-6.910	-4.97	-5.375	-4.850	-5.165	-3.967	-4.235	-4.909	-4.346
NC9	-5.932	-6.022	-5.254	-6.528	-4.92	-5.095	-4.802	-4.977	-3.958	-4.431	-4.967	-4.342
NC10	-4.174	-5.298	-3.401	-4.381	-3.40	-4.196	-4.561	-4.319	-4.365	-3.268	-3.110	-3.811
NC11	-6.523	-8.385	-7.017	-6.929	-5.87	-5.085	-4.526	-4.941	-5.252	-4.537	-4.764	-4.649
NC12	-3.824	-3.159	-4.174	-4.213	-4.01	-3.713	-3.669	-3.636	-3.981	-3.826	-3.734	-3.409
NC13	-4.208	-4.763	-5.412	-5.445	-4.06	-4.072	-3.934	-4.262	-3.907	-4.229	-4.500	-4.295
NC14	-5.301	-5.479	-5.663	-5.752	-4.36	-3.867	-4.146	-4.150	-3.800	-4.570	-4.291	-4.735
NC15	-4.046	-3.354	-4.633	-4.568	-3.05	-3.312	-3.068	-3.099	-3.961	-3.865	-3.940	-3.905
NC16	-3.047	-2.636	-4.012	-4.105	-4.82	-2.163	-2.336	-2.209	-2.746	-2.597	-2.943	-3.544
NC17	-3.048	-2.434	-3.628	-3.807	-4.44	-1.631	-1.737	-1.884	-2.851	-2.094	-2.617	-3.241
NC18	-3.641	-4.915	-4.504	-5.090	-3.97	-3.161	-2.568	-3.497	-3.279	-3.329	-3.583	-4.068
NC19	-3.630	-4.510	-3.412	-4.155	-3.99	-3.381	-2.656	-3.652	-3.270	-3.703	-3.301	-3.881
NC20	-3.748	-6.207	-4.623	-6.528	-4.91	-5.016	-5.120	-4.903	-3.873	-4.339	-4.844	-4.375
NC21	-4.131	-4.686	-5.571	-4.967	-3.58	-4.239	-3.860	-4.440	-4.341	-4.331	-4.580	-2.702
NC22	-4.790	-5.281	-4.379	-5.126	-4.01	-4.641	-4.740	-4.490	-3.497	-3.912	-4.537	-3.949
NC23	-4.866	-4.967	-5.003	-6.146	-4.30	-5.390	-5.152	-5.265	-4.046	-4.224	-4.817	-4.404
NC24	-5.301	-4.763	-5.400	-5.445	-4.06	-4.072	-3.934	-4.262	-3.907	-4.229	-4.500	-4.295
NC25	-5.301	-6.152	-6.970	-6.442	-4.65	-4.480	-6.256	-4.603	-4.375	-4.136	-4.808	-4.840
NC26	-5.301	-4.871	-5.239	-5.135	-4.00	-4.246	-4.250	-4.247	-4.147	-4.145	-3.626	-3.743
NC27	-5.301	-5.121	-6.803	-5.884	-4.36	-4.383	-5.040	-4.497	-4.545	-4.362	-4.177	-4.273
NC28	-5.301	-5.417	-6.364	-6.013	-4.30	-4.394	-3.605	-4.553	-4.390	-4.214	-4.425	-4.484
NC29	-5.163	-6.207	-5.423	-6.528	-4.94	-5.016	-5.120	-4.903	-3.873	-4.339	-4.844	-4.375
NC30	-5.019	-5.152	-5.003	-6.146	-4.30	-5.390	-5.152	-5.265	-4.046	-4.224	-4.817	-4.404
NC31	-5.126	-6.502	-5.759	-6.910	-4.97	-5.288	-5.250	-5.082	-3.897	-4.256	-5.111	-4.428
NC32	-4.398	-4.391	-4.822	-4.939	-4.02	-4.036	-4.235	-3.684	-4.204	-4.287	-3.902	-3.881
NC33	-5.163	-6.175	-5.504	-6.528	-4.93	-5.057	-4.972	-4.932	-3.988	-4.434	-4.935	-4.395
NC34	-2.609	-1.946	-3.927	-3.931	-3.17	-1.465	-2.144	-1.326	-3.267	-2.497	-3.017	-3.152
NC35	-5.210	-6.941	-6.028	-7.260	-5.21	-5.554	-5.682	-5.201	-4.747	-5.215	-5.283	-5.611
NC36	-5.197	-5.192	-5.331	-6.234	-4.01	-4.750	-4.941	-4.510	-3.975	-4.188	-4.592	-2.906
NC37	-5.299	-5.152	-5.051	-6.146	-4.30	-5.504	-4.769	-5.383	-4.109	-4.277	-4.959	-4.368

NC38	-3.719	-4.307	-4.873	-5.068	-4.56	-3.571	-3.384	-3.241	-3.160	-3.476	-3.246	-4.048
NC39	-5.511	-6.502	-5.759	-6.910	-4.97	-5.288	-5.250	-5.082	-3.897	-4.256	-5.111	-4.428
NC40	-3.770	-4.653	-4.616	-4.659	-4.55	-2.914	-3.829	-2.850	-3.432	-3.464	-2.660	-3.301
NC41	-5.473	-5.062	-4.249	-5.269	-3.85	-4.802	-4.499	-4.700	-4.134	-4.109	-5.477	-3.741
NC42	-3.783	-4.343	-6.201	-5.589	-4.06	-4.522	-4.052	-4.772	-4.562	-4.826	-4.823	-3.612
NC43	-4.466	-6.377	-6.137	-6.469	-4.64	-4.587	-4.737	-4.983	-4.573	-4.536	-4.302	-5.178
NC44	-3.810	-4.660	-5.094	-4.746	-3.97	-4.088	-3.467	-4.315	-4.403	-4.160	-3.811	-4.362
NC45	-3.824	-3.739	-5.410	-4.783	-3.63	-4.265	-3.408	-4.417	-4.251	-4.308	-4.271	-3.770
NC46	-4.393	-5.281	-4.381	-5.126	-4.01	-4.641	-4.740	-4.490	-3.497	-3.912	-4.537	-3.949
NC47	-2.832	-2.291	-4.009	-4.092	-4.26	-1.338	-1.500	-1.287	-2.194	-2.190	-2.351	-3.238
NC48	-4.451	-4.408	-3.966	-4.821	-3.78	-4.511	-4.986	-4.890	-4.354	-4.204	-4.766	-4.717
NC49	-3.450	-4.915	-4.505	-5.090	-3.97	-3.161	-2.568	-3.497	-3.279	-3.329	-3.583	-4.068
NC50	-4.547	-6.176	-5.824	-6.691	-5.21	-4.762	-5.051	-5.205	-4.577	-4.492	-4.608	-5.207
NC51	-4.886	-4.304	-5.820	-5.098	-3.94	-4.113	-4.304	-4.200	-4.355	-4.215	-3.861	-3.737
NC52	-2.892	-2.292	-3.712	-3.871	-3.52	-2.082	-2.716	-2.017	-3.591	-2.826	-3.526	-3.373
NC53	-3.893	-4.650	-4.352	-4.451	-4.47	-3.234	-3.305	-2.941	-3.231	-3.295	-3.292	-3.705
NC54	-4.459	-4.820	-3.978	-5.138	-4.29	-3.954	-2.717	-3.896	-3.052	-3.438	-2.904	-2.885
NC55	-4.527	-5.312	-4.295	-5.126	-4.05	-4.583	-4.914	-4.436	-3.340	-3.991	-4.427	-3.689
NC56	-5.000	-4.558	-4.663	-4.748	-4.04	-3.591	-3.632	-3.650	-4.308	-4.015	-3.391	-4.662
NC57	-5.442	-6.594	-5.387	-6.364	-4.55	-5.069	-5.297	-4.977	-3.876	-4.258	-4.780	-4.151
NC58	-5.464	-6.175	-5.504	-6.528	-4.93	-5.057	-4.972	-4.932	-3.988	-4.434	-4.935	-4.395
NC59	-4.349	-4.609	-4.444	-4.654	-3.60	-4.284	-4.650	-4.580	-4.365	-4.064	-4.571	-4.673
NC60	-5.527	-6.941	-6.022	-7.260	-5.21	-5.554	-5.682	-5.201	-4.747	-5.215	-5.283	-5.611
NC61	-3.774	-3.022	-4.644	-4.554	-5.01	-2.355	-2.208	-2.484	-2.518	-2.798	-3.890	-3.242
NC62	-6.069	-5.957	-5.429	-6.671	-4.83	-5.246	-4.672	-5.002	-3.956	-4.599	-5.142	-4.430

[a]LogS_exp: The experimental aqueous solubility values in logarithmic units of mol/L. [b]MOE V2016.0802. [c]QikProp 2018-4 QP18. [d]QikProp 2018-4 CIQP18. [e]AlogGPS V2.1. [f]4-layer DNN model. [g]1-layer DNN model. [h]6-layer DNN model. [i]8-layer ResNet-like model. [j]14-layer ResNet-like model. [k]20-layer ResNet-like model. [l]26-layer ResNet-like model.

Supplementary Table S6. The mean, standard deviation and 95% confidence interval of R^2 and RMSE values for 10,000 bootstrap sampling of the established tools, the shallow-net and deeper-net deep learning models on 62 recently-published novel compounds

	R^2			RMSE		
**Model**	mean	std	CI (95%)[a]	mean	std	CI (95%)[a]
Established tools						
MOE V2016.0802	-0.089	0.261	(-0.094, -0.083)	0.904	0.078	(0.902, 0.905)
QikProp 2018-4 QP18	-0.137	0.273	(-0.142, -0.131)	0.923	0.078	(0.922, 0.925)
QikProp 2018-4 CIQP18	-0.790	0.394	(-0.797, -0.782)	1.160	0.080	(1.158, 1.161)
AlogGPS V2.1	0.131	0.138	(0.129, 0.134)	0.812	0.057	(0.811, 0.813)
Shallow-net deep learning model of a typically-employed architecture for solubility prediction						
4-layer DNN model	0.285	0.124	(0.283, 0.288)	0.737	0.061	(0.736, 0.738)
Shallow-net deep learning models developed in this work						
1-layer DNN model	0.054	0.189	(0.050, 0.057)	0.846	0.072	(0.845, 0.847)
6-layer DNN model	0.242	0.123	(0.239, 0.244)	0.760	0.058	(0.759, 0.761)
8-layer ResNet-like model	-0.272	0.283	(-0.277, -0.266)	0.978	0.080	(0.977, 0.980)
Shallow-net deep learning models developed in this work						
14-layer ResNet-like model	0.103	0.171	(0.099, 0.106)	0.824	0.063	(0.823, 0.825)
20-layer ResNet-like model	**0.390**	**0.144**	**(0.387, 0.393)**	**0.677**	**0.065**	**(0.676, 0.679)**
26-layer ResNet-like model	0.042	0.208	(0.038, 0.046)	0.850	0.074	(0.848, 0.851)

[a] 95% confidence interval.

2 Supplementary Methods

Supplementary Method M1. The synthetic methods of compound SC5 and SC6

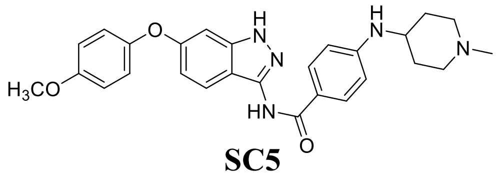

SC5

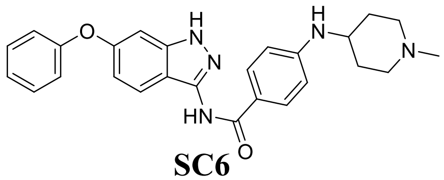

SC6

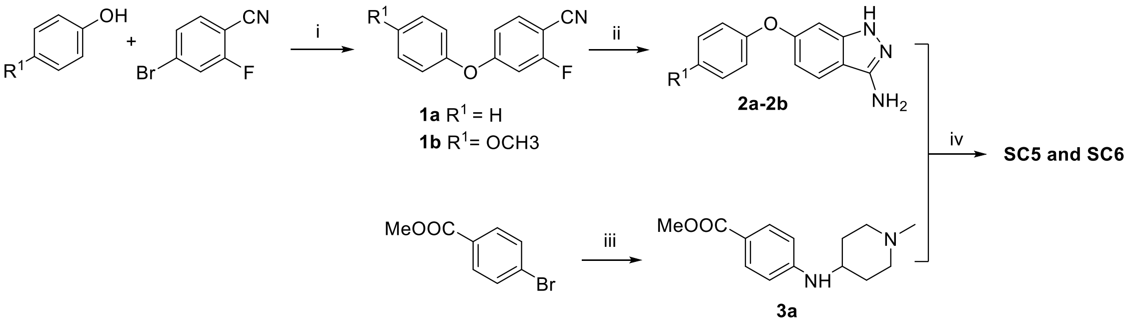

Reagents and conditions: (i) $Pd(OAc)_2$, 564483-19-8, K_3PO_4, toluene, 100 ^oC; (ii) $NH_2NH_2·H_2O$, EtOH, reflux; (iii) 1-methylpiperidine-4-amine, $Pd(OAc)_2$, BINAP, Cs_2CO_3, toluene, 100 ^oC; (iv) $Al(CH_3)_3$, toluene, 90 ^oC.

General procedure for the synthesis of compound **1a-1b**

Phenol (0.282 g, 3 mmol), 4-bromo-2-fluorobenzonitrile (0.5 g, 2.5 mmol), $Pd(OAc)_2$ (0.112 g, 0.05 mmol), 564483-19-8 (0.318 g, 0.075 mmol), K_3PO_4 (1.06 g, 5 mmol) and anhydrous toluene were added to a 100 mL flask under nitrogen atmosphere. The solution was stirred at 100 ^oC for 24 h. Then, the reaction mixture was filtered. The solvent was removed under reduced pressure and purified by column chromatography (PE : EA= 20 : 1) to afford compound **1a** as an oil. Yield: 92.8%. ^1H NMR (300 MHz, $CDCl_3$) δ 7.53 (dd, *J* = 8.7, 7.4 Hz, 1H), 7.48-7.40 (m, 2H), 7.31-7.24 (m, 1H), 7.11-7.05 (m, 2H), 6.81 (ddd, *J* = 8.7, 2.4, 0.7 Hz, 1H), 6.72 (dd, *J* = 10.5, 2.4 Hz, 1H). MS (m/z): $[M+Na]^+$ 236.0.

The synthesis of compound **1b**

The preparation of compound **1b** was similar with that of compound **1a** to afford title compound as an oil. Yield: 78.4%. ^1H NMR (300 MHz, DMSO-d_6) δ 7.87 (dd, *J* = 8.8, 7.8 Hz, 1H), 7.21-7.10 (m, 2H), 7.10-6.99 (m, 3H), 6.85 (ddd, *J* = 8.8, 2.4, 0.6 Hz, 1H), 3.78 (s, 3H).

General procedure for the synthesis of compound **2a-2b**

Compound **1a** (0.213 g, 1 mmol), 80% hydrazine hydrate (0.15 g, 1.5 mmol) and 10 mL of ethanol were added to a 50 mL flask. The solution was heated to reflux for 5 h. After cooling, the solvent was removed under reduced pressure, and ice water (20 mL) was added. The mixture was extracted with ethyl acetate (20 mL × 3). The combined organic layers were washed twice with saturated brine (20 mL × 2) and dried over anhydrous $MgSO_4$. The desiccant was filtered out, and the filtrate was concentrated under reduced pressure. The residue was purified by column chromatography (PE : EA = 2 : 1) to afford **2a** as a yellow solid. Yield: 47.3%. m.p.:154-156 ^oC. ^1H NMR (300 MHz, $CDCl_3$) δ 7.42 (dd, *J* = 8.6, 0.7 Hz, 1H), 7.30-7.25 (m, 2H), 7.11-7.04 (m, 1H), 7.00-6.94 (m, 2H), 6.77-6.70 (m, 2H). MS (m/z): $[M+H]^+$ 226.1.

The synthesis of compound **2b**

The preparation of compound **2b** was similar with that of compound **2a** to afford title compound as a yellow solid. Yield: 62.8%. m.p.: 172-174 °C. ^1H NMR (300 MHz, DMSO-d_6) δ 11.13 (s, 1H), 7.62 (d, *J* = 8.7 Hz, 1H), 7.08-7.00 (m, 2H), 7.00-6.93 (m, 2H), 6.61 (dd, *J* = 8.7, 2.1 Hz, 1H), 6.50 (dd, *J* = 2.1, 0.6 Hz, 1H), 5.30 (s, 2H), 3.76 (s, 3H).

The synthesis of compound **3a**

Methyl 4-bromobenzoate (0.43 g, 2 mmol), 1-methylpiperidin-4-amine (0.23 g, 2 mmol), $Pd(OAc)_2$ (0.22 g, 0.1 mmol), BINAP (0.62 g, 0.1 mmol), Cs_2CO_3 (1.37 g, 2.2 mmol), anhydrous toluene (15 mL) were added to a 100 mL two-neck flask under argon atmosphere. The solution was stirred at 90 °C for 12 h, followed by quenched with ice water (20 mL). The mixture was extracted with ethyl acetate (40 mL × 3). The combined organic phases were concentrated under reduced pressure and purified by column chromatography (EA: MeOH = 20 : 1) to afford **3a** as a pink oil. Yield: 49.6%. ^1H NMR (300 MHz, DMSO-d_6) δ 7.67 (d, *J* = 8.8 Hz, 2H), 6.60 (d, *J* = 8.8 Hz, 2H), 6.46 (d, *J* = 7.7 Hz, 1H), 3.73 (s,

3H), 3.33 (d, J = 3.9 Hz, 1H), 2.93-2.82 (m, 2H), 2.29 (s, 3H), 2.27-2.17 (m, 2H), 1.96-1.85 (m, 2H), 1.55-1.42 (m, 2H). MS (m/z): $[M+H]^+$ 249.1.

N-(6-(4-Methoxyphenoxy)-1H-indazol-3-yl)-4-((1-methylpiperidin-4-yl)amino)benzamide (**SC5**)

The preparation of compound **4b** was similar with that of compound **4a** to afford title compound as a white solid. Yield: 27.1%. m.p.: 236-238 °C. ^1H NMR (300 MHz, Methanol-d_4) δ 7.87 (s, 2H), 7.75 (s, 1H), 7.04 (s, 2H), 6.97 (s, 2H), 6.85 (s, 1H), 6.80 (s, 1H), 6.71 (s, 2H), 3.81 (s, 3H), 3.50 (s, 1H), 3.05 (s, 2H), 2.51 (s, 2H), 2.46 (s, 3H), 2.11 (s, 2H), 1.66 (s, 2H). MS (m/z): $[M+H]^+$ 472.5.

4-((1-methylpiperidin-4-yl)amino)-N-(6-phenoxy-1H-indazol-3-yl)benzamide (**SC6**)

Compound **2a** (0.225 g, 1 mmol), compound **3a** (0.273 g, 1.1 mmol), 2 M trimethylaluminum toluene solution (2 mL, 4 mmol) and anhydrous toluene (5 mL) were added to a 50 mL two-neck flask under argon atmosphere. The solution was stirred at 90 °C for 10 h, followed by quenched with anhydrous ethanol (20 mL). The solvent was concentrated under reduced pressure and purified by column chromatography (EA : MeOH = 20 : 1) to afford **4a** as a white solid. Yield: 27.2%. m.p.: 126-128 °C. ^1H NMR (300 MHz, Methanol-d_4) δ 7.75 (s, 2H), 7.67 (s, 1H), 7.28 (s, 2H), 7.05 (s, 1H), 6.95 (s, 2H), 6.80 (s, 1H), 6.76 (d, J = 0.5 Hz, 1H), 6.59 (s, 2H), 3.34 (s, 1H), 2.82 (s, 2H), 2.27 (s, 3H), 2.23 (s, 2H), 1.95 (s, 2H), 1.50 (s, 2H). MS (m/z): $[M+H]^+$ 442.2.

REFERENCES

Theppawong, A., Van de Walle, T., Grootaert, C., Bultinck, M., Desmet, T., Van Camp, J., and D'Hooghe, M. (2018). Synthesis of Novel Aza-aromatic Curcuminoids with Improved Biological Activities towards Various Cancer Cell Lines. *ChemistryOpen* 7, 381-392. doi: 10.1002/open.201800029

Wilson, C. R., Gessner, R. K., Moosa, A., Seldon, R., Warner, D. F., Mizrahi, V., et al. (2017). Novel Antitubercular 6-Dialkylaminopyrimidine Carboxamides from Phenotypic Whole-Cell High Throughput Screening of a SoftFocus Library: Structure-Activity Relationship and Target Identification Studies. *J. Med. Chem.* 60, 10118-10134. doi: 10.1021/acs.jmedchem.7b01347.

Thompson, A. M., O'Connor, P. D., Marshall, A. J., Blaser, A., Yardley, V., Maes, L., et al. (2018). Development of (6 R)-2-Nitro-6-[4-(trifluoromethoxy)phenoxy]-6,7-dihydro-5 H-imidazo[2,1-b][1,3]oxazine (DNDI-8219): A New Lead for Visceral Leishmaniasis. *J. Med. Chem.* 61, 2329-2352. doi: 10.1021/acs.jmedchem.7b01581.

Hamdy, A. K., Sheha, M. M., Abdel-Hafez, A. A., and Shouman, S. A. (2017). Design, Synthesis, and Cytotoxicity Evaluation of Novel Griseofulvin Analogues with Improved Water Solubility. *Int. J. Med. Chem.* 2017, 7386125. doi: 10.1155/2017/7386125.

Ortiz, D., Guiguemde, W. A., Hammill, J. T., Carrillo, A. K., Chen, Y., Connelly, M., et al. (2017). Discovery of novel, orally bioavailable, antileishmanial compounds using phenotypic screening. *PLoS Negl. Trop. Dis.* 11, e0006157. doi: 10.1371/journal.pntd.0006157.
