# Supplementary figures and images for: Improved Prediction of Aqueous Solubility of Novel Compounds by Going Deeper With Deep Learning
Source: Front Oncol. 2020 Feb 11;10:121. doi: 10.3389/fonc.2020.00121 (PMC7026387; doi:10.3389/fonc.2020.00121)

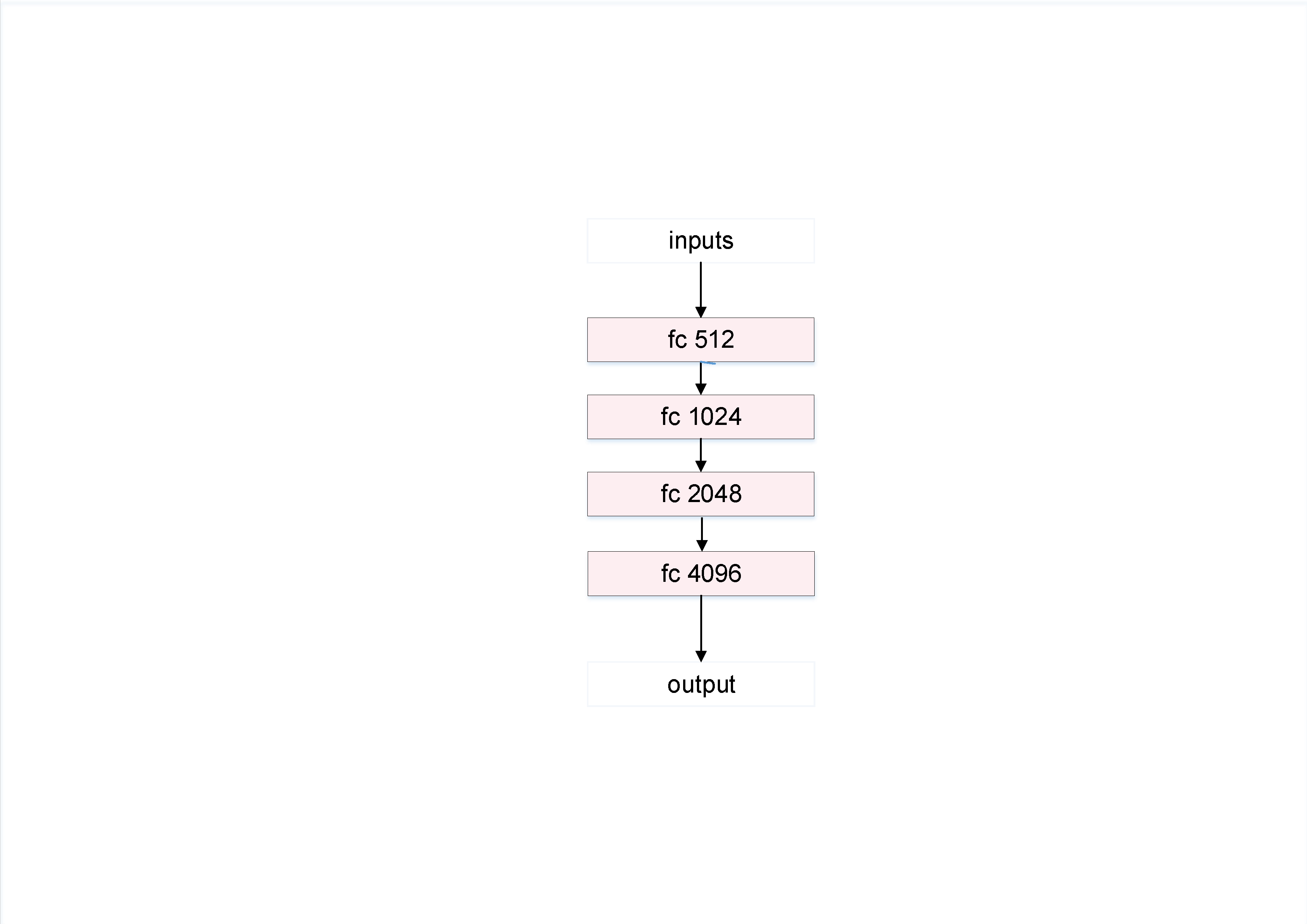

Supplement: Supplementary file 1 [file Data_Sheet_1.ZIP › Supplementary Material/Supplementary Figure S2. The architecture of the 4-layer DNN model.tiff]

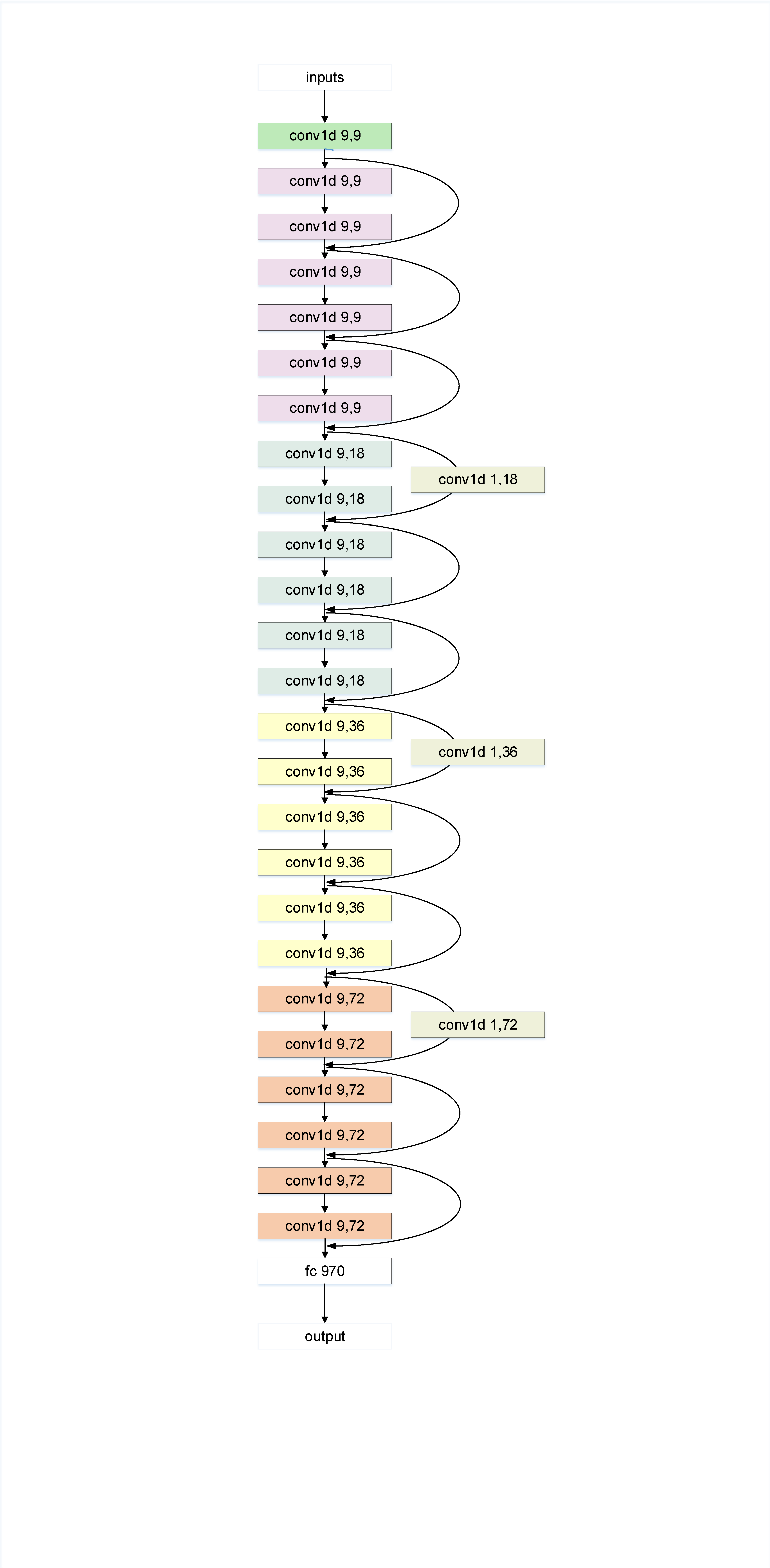

Supplement: Supplementary file 1 [file Data_Sheet_1.ZIP › Supplementary Material/Supplementary Figure S3. The architecture of the 26-layer CNN ResNet-like deep learning model.tiff]
